# Descriptive comparative morphology of the eye and ocular adnexa in Felidae: observations from a clouded leopard (*Neofelis nebulosa*), African lion (*Panthera leo*), and Pallas’s cat (*Otocolobus manul*)

**DOI:** 10.1186/s12917-026-05480-1

**Published:** 2026-04-22

**Authors:** Joanna Klećkowska-Nawrot, Shaw Badenhorst, Wojciech Borawski, Karolina Goździewska-Harłajczuk, Abit Aktaş, Aleksander Chrószcz

**Affiliations:** 1 Department of Biostructure and Animal Physiology, Division of Animal Anatomy, Faculty of Veterinary Medicine, Wrocław University of Environmental and Life Sciences, 1 Kożuchowska Street, Wrocław, 51-631 Poland; 2 Evolutionary Studies Institute, University of the Witwatersrand, 1 Jan Smuts Ave, Braamfontein, Johannesburg, 2000 South Africa; 3 Department and Clinic of Surgery, Faculty of Veterinary Medicine, Wrocław University of Environmental and Life Sciences, 51 Grunwaldzki Square, Wrocław, 50- 366 Poland; 4 Department of Histology and Embryology, Faculty of Veterinary Medicine, Istanbul University-Cerrahpasa, Istanbul, Turkey

**Keywords:** Accessory organs of the eye, African lion, Angola lion, Clouded leopard, Eyeball, Orbital region, Pallas’s cat, Vision

## Abstract

**Background:**

The Felidae family includes diverse species with marked variation in ocular morphology. This study describes and qualitatively compares ocular morphology in a limited number of specimens: the clouded leopard (*Neofelis nebulosa*, *n* = 1), the African lion (*Panthera leo*, including two Angola lions, *P. l. bleyenberghi*, *n* = 2), and Pallas’s cat (*Otocolobus manul*, including *O. m. manul*, *n* = 1), whereas morphometric assessment of the orbital region was additionally based on skull material (clouded leopard, *n* = 1; Pallas’s cat, *n* = 2; lions, *n* = 11).

**Materials and methods:**

Morphometric analyses were performed for the orbital region, eyeball, and selected accessory organs of the eye, using combined macroscopic and microscopic examinations. Histological staining included Mayer’s hematoxylin and eosin, Heidenhain’s Azan trichrome, Movat pentachrome, Masson-Goldner trichrome, Mallory’s trichrome with aniline blue, and Masson-Fontana, as well as histochemical staining with PAS, alcian blue (pH 1.0 and 2.5), alcian blue pH 2.5/PAS, and colloidal iron. Because of the very limited number of specimens, the study was strictly descriptive, and no statistical comparisons were performed.

**Results:**

In the examined specimens, eyeball size generally corresponded to animal body size, whereas eyeball shape varied among individuals: the examined Angola lions had elliptical eyeballs, the examined clouded leopard had a nearly round eyeball, and the examined Pallas’s cat had a slightly elliptical eyeball. Corneal size and shape varied among the examined individuals. Histologically, the cornea consisted of four layers, Bowman’s layer was absent, and epithelial thickness varied among specimens. Palisades of Vogt were identified in the examined material. The tapetum lucidum varied in size among the examined animals and was most pronounced in the Angola lions; its coloration ranged from milky blue to blue-green. The anterior chamber depth appeared to correspond with eyeball size. Pupil shape also differed among the examined animals. Histological and histochemical analyses of the ocular adnexa revealed interindividual and intertaxon differences in microstructure; however, the very limited material did not allow broader interpretation. Orbital morphometric values generally followed overall skull size. Possible sex-related variation was observed in the lion material, but this requires verification in a larger series.

**Conclusion:**

The present study provides a descriptive anatomical and histological characterization of the eye and selected ocular adnexa in a small number of representatives of three felid taxa. These findings may serve as baseline reference data for comparative anatomy and veterinary ophthalmology, but they should not be interpreted as species-level patterns.

**Limitations:**

Due to the limited sample size, these observations are preliminary and do not allow for species-level or population-level inferences. Because ante-mortem ophthalmic examinations were not available, subclinical ocular abnormalities cannot be excluded. In addition, because the material was collected post-mortem and subjected to fixation, processing, and sectioning, some morphometric values may have been affected by tissue distortion or shrinkage. Further studies based on larger series and standardized clinical evaluation are needed to verify the patterns observed in the present material.

**Supplementary Information:**

The online version contains supplementary material available at 10.1186/s12917-026-05480-1.

## Background

Felids are highly specialized predators in the animal kingdom. They use vision to detect prey and to navigate their surroundings, as well as for intraspecific communication, including in domestic cats, making it one of their most important senses alongside hearing [1]. When a cat, such as an Angola lion (*Panthera leo bleyenberghi*) or a Pallas’s cat (*Otocolobus manul*), stalks its prey, it must accurately determine the distance between itself and its potential prey [2]. Each step closer to the target increases the chances of a successful hunt. Among the five senses that mediate an individual’s interaction with the environment, vision is especially important in Felidae, including arboreal felids, such as the clouded leopard (*Neofelis nebulosa*), which moves among tree branches and leaps from one to another [3]. Every movement must be precise to avoid falling. This accuracy is possible thanks to binocular vision, which is highly developed in Felidae compared with other carnivores [4]. Therefore, the comparative morphology of the eyeball and the anatomy of the accessory organs of the eye can be crucial for understanding visual function in felids. There are several other adaptations that enhance cats’ sense of vision. One of the most significant is the size and shape of the pupil. However, as pupil size increases, a larger lens is required to prevent light from being distorted at the edges when passing through. Additionally, the curvature of both the lens and cornea must also increase [5]. The available literature describes the pupil shape and its functional significance [2]. Pantherinae, such as the lion and the clouded leopard, have broadly elliptical pupils [2]. The same is true for the much smaller Pallas’s cat. It is often suggested that this pupil shape is typical of animals active during daylight, but studies have confirmed that lions primarily hunt at dusk, dawn, and during the night [6]. The opposite is generally assumed for the clouded leopard, which is considered a crepuscular or nocturnal species. However, recent observations have suggested that clouded leopards may also be active during the day [7]. The pattern of daily activity in a cat depends on multiple factors, including human disturbance, season, temperature, prey activity, and whether the cat itself is being hunted [8]. Accordingly, direct links between behavior and ocular morphology may be complex and are not fully established. Like most carnivores, with a few exceptions, cats possess an additional layer called the tapetum lucidum, which enhances visual sensitivity in low-light conditions by reflecting light back through the retina [9]. This feature is considered important for low-light vision in many felids. Although representatives of this family of mammals are mostly solitary – except for lions and, in some cases, also the cheetah (*Acinonyx jubatus*) – they possess a complex system of visual communication [10]. Their bodies are highly expressive, particularly their heads, where the ears, teeth, lips, whiskers, and eyes convey complex information. For example, ear position and facial expressions can signal aggression or appeasement, whereas tail position and body posture can indicate arousal or intent during social encounters [1, 11]. Many of these features are accentuated by contrasting markings. Thus, vision contributes not only to predation, but also to intraspecific signaling and social interactions.

Most studies describing the typical structure of the eyeball and accessory eye organs at both macroscopic and microscopic levels, as well as ophthalmological cases in Felidae, focus on the domestic cat (*Felis catus*) (Table 1 and Table S1). However, there are very few studies on the anatomy of the visual system in wild felids (Table 1 and Table S1). There is considerably more information available from clinical case reports discussing diseases of the eyeball and ocular adnexa in wild felids [12–30], as these animals are frequent inhabitants of zoological gardens (Table 1 and Table S1). Therefore, a detailed anatomical description of the eye and ocular adnexa in wild felids is needed, not only as a contribution to the morphological sciences, but also as a source of valuable information for clinicians and biologists.

**Table 1:** Literature summary for study taxa

SPECIES	MORPHOLOGY					OPHTHALMOLOGICAL EXAMINATION AND PATHOLOGY	LITERATURE
	ORBITAL REGION/ EYE TUNICS	UPPER and LOWER EYELIDS	SUPERFICIAL GLAND OF THE THIRD EYELID	THIRD EYELID	LACRIMAL GLAND		
African lion (*Panthera leo* Linnaeus, 1758)	+ (orbital region – foramina, cornea)	–	–	–	–	+	[12–26]
Angola lion (*Panthera leo bleyenberghi* Lönnberg, 1914)	–	–	–	–	–	+	[27, 28]
Clouded leopard (*Neofelis nebulosa* Griffith, 1821)	+ (orbital region –foramina)	–	–	–	–	+	[19, 29, 30]
Pallas’s cat (*Otocolobus manul manul* Pallas, 1776)	–	–	–	–	–	–	–

The aim of this study was to provide a detailed descriptive account of the anatomy of the orbit, eyeball, and selected structures of the visual system in three species of felids: the clouded leopard, the Pallas’s cat, and the African lion. Due to the limited number of specimens available for study, the comparisons are primarily descriptive. This analysis seeks to describe observed similarities and differences among the examined specimens (including subspecies) and to place these findings in the context of the published literature on Felidae and other terrestrial carnivores. Because of the limited material, the present study was not designed to evaluate species-level patterns, ecological adaptations, or functional relationships. Instead, it provides descriptive observations from the examined specimens and compares them cautiously with previous reports. Full literature overview [12, 18, 19, 27, 31–79] was listed in Supplementary Table S1.

## Materials and methods

An important practical aspect of this research is its relevance to veterinary medicine. A more detailed understanding of the ocular anatomy in wild felids may support the diagnosis and treatment of eye disorders, both in captivity and in the wild. Because the material was obtained post-mortem and accessible veterinary records did not include any ante-mortem ophthalmic examinations, the findings should be interpreted as descriptive observations rather than normative reference values. The time between animal death and sample collection did not exceed 2 h. The veterinary records and post-mortem gross examination did not reveal any macroscopic abnormalities of the head or eyes; nevertheless, subclinical ocular changes cannot be excluded. Accordingly, we present these data as an anatomical and histological description that may assist clinicians and support future studies using larger, standardized cohorts.

### Specimens

**Fig. 1:**
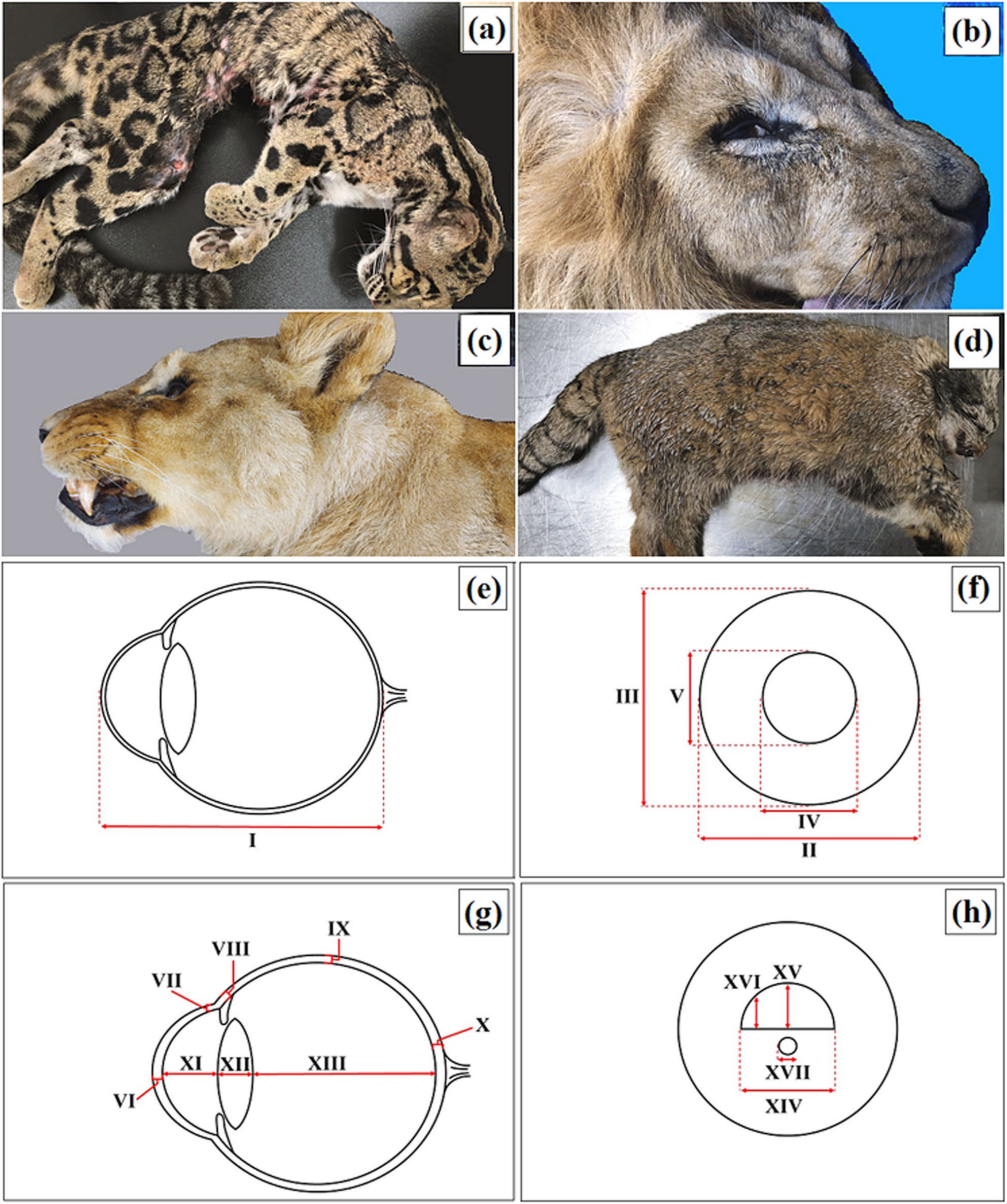
Study specimens and ocular measurement scheme. Macrographs of the examined clouded leopard (*Neofelis nebulosa*) (**a**), Angola lions (*Panthera leo bleyenberghi*) (**b**, **c**), and Pallas’s cat (*Otocolobus manul manul*) (**d**). Panels (**e–h**) illustrate the ocular measurement scheme used for the enucleated globes. External globe and corneal diameters were measured on intact eyes prior to sectioning, whereas internal dimensions were measured immediately after a standardized section through the optical axis. Abbreviations, definitions, and anatomical landmarks are provided in Table 3.

The studied material consisted of eyeballs and selected ocular adnexa from four individuals representing three felid taxa: the clouded leopard (*n* = 1), the Angola lion (*n* = 2), and the Pallas’s cat (*Otocolobus manul manul*) (*n* = 1). Specimen metadata (sex, age, body measurements, source, cause of death, and post-mortem interval) are summarized in Table 2, and representative images are shown in Fig. 1a and d.

**Table 2:** Specimen metadata for eyeball and ocular adnexa analyses

SPECIES	SEX	DATE OF BIRTH	AGE AT DEATH	BODY MASS (KG)	BODY LENGTH W/O TAIL (CM)	TAIL LENGTH (CM)	SOURCE	YEAR COLLECTED	CAUSE OF DEATH	PMI (h)	ANTE-MORTEM OPHTHALMIC EXAM	GROSS OCULAR ABNORMALITIES (POST-MORTEM)
Clouded leopard (*Neofelis nebulosa*)	F	2014-03-17	5 y 6 mo 24 d	11.2	69.0	62.0	Wrocław Zoo	2019–2022	male attack during pairing	≤ 2	No	None reported
Angola lion (*Panthera leo bleyenberghi*)	F	2006-6-17	14 y 9 mo 28 d	105.0	146.0	71.0	Wrocław Zoo	2019–2022	natural death due to old age	≤ 2	No	None reported
Angola lion (*Panthera leo bleyenberghi*)	M	2007-6-17	13 y 3 mo 14 d	120.0	189.0	78.0	Wrocław Zoo	2019–2022	failure to recover from a veterinary procedure	≤ 2	No	None reported
Pallas’s cat (*Otocolobus manul manul*)	M	2016-4-21	4 y 6 mo 8 d	5.1	47.0	23.0	Wrocław Zoo	2019–2022	glomerulonephritis	≤ 2	No	None reported

PMI, *Post-mortem* interval (time from death to tissue collection)

None of the animals were euthanized for the purpose of this study

**Table 3:** Definitions of ocular measurements and measurement conditions

ABBREV.	MEASUREMENT (UNIT)	DEFINITION/ ANATOMICAL LANDMARKS	CONDITION (INTACT VS SECTIONED)	TOOL / METHOD
AED	Axial eye diameter (mm)	Anterior corneal surface to optic disc along optical axis	Intact (before sectioning)	Digital caliper
MaEED	Max equatorial eye diameter (mm)	Maximum transverse diameter at equator	Intact	Digital caliper
MiEED	Min equatorial eye diameter (mm)	Minimum transverse diameter at equator	Intact	Digital caliper
MaCD	Max corneal diameter (mm)	Maximum corneal diameter (limbus to limbus)	Intact	Digital caliper
MiCD	Min corneal diameter (mm)	Minimum corneal diameter perpendicular to MaCD	Intact	Digital caliper
CAT	Corneal axial thickness (mm)	Thickness at central cornea	Sectioned	Digital caliper
CPT	Corneal peripheral thickness (mm)	Thickness at peripheral cornea at the limbus	Sectioned	Digital caliper
SLT	Scleral limbus thickness (mm)	Sclera thickness at limbus	Sectioned	Digital caliper
SET	Scleral equator thickness (mm)	Sclera thickness at equator	Sectioned	Digital caliper
SONT	Scleral optic nerve thickness (mm)	Sclera thickness at optic nerve disc	Sectioned	Digital caliper
AACD	Anterior chamber depth (mm)	Posterior corneal surface to anterior lens surface along optical axis	Sectioned	Digital caliper
LAL	Lens axial length (mm)	Anterior to posterior pole of lens	Sectioned	Digital caliper
VCD	Vitreous chamber depth (mm)	Posterior lens surface to optic disc along optical axis	Sectioned	Digital caliper
TLL	Tapetum lucidum length (mm)	Long axis of tapetum	Sectioned	Digital caliper
TLW1	Tapetum lucidum width 1 (mm)	Width at widest point	Sectioned	Digital caliper
TLW2	Tapetum lucidum width 2 (mm)	Width at narrowest point	Sectioned	Digital caliper
OND	Optic nerve diameter (mm)	Diameter of optic nerve at optic nerve stump	Sectioned	Digital caliper
CRW	Ciliary ring width (mm)	Width of ciliary ring at the temporal quadrant	Sectioned	Digital caliper
CPL	Ciliary processes length (mm)	Length of ciliary processes on the ciliary crown	Sectioned	Digital caliper

These animals were housed at the Wrocław Zoological Garden (Poland), and the cadavers were collected between 2019 and 2022. None of the animals were euthanized for this study; they were obtained after natural death. Immediately after collection, the eyeballs and ocular adnexa were examined grossly for damage or overt abnormalities and were processed as described below; no specimens were excluded based on gross examination. The specimens are stored in the Division of Animal Anatomy, Department of Biostructure and Animal Physiology, Wrocław University of Environmental and Life Sciences (Poland).

Many authors subsume the Angola lion under *Panthera leo leo* [80], however, we use the designation *Panthera leo bleyenberghi* in accordance with the identification records provided by the Wrocław Zoological Garden (Poland). For clarity, eyeball and ocular adnexa analyses were based on these four individuals. For the anatomical cohort, tissues were fixed in 4% buffered formaldehyde (see Anatomical analysis) and subsequently stored in fixative until processing for histology.

The anatomical description and morphometry of the orbital region were based on skulls from the following specimens: one clouded leopard – FE/NN/1, two Pallas’s cats (including the subspecies O. *m. manul*) – FE/OM/1, FE/OMM/2, and eleven lions (*Panthera leo*, including two Angola lions) – FE/PL/1, FE/PL/2, BPI/C/183, BPI/C/184, BPI/C/185, BPI/C/186, BPI/C/187, ZA17, BPI/4/1351, and FE/PLB/1, FE/PLB/2. FE/NN/1 was a female, and FE/OMM/2 was a male, both from Wrocław Zoological Garden (Poland). FE/OM/1 (from the Czech Republic) was a male. Among the Angola lions, FE/PLB/1 was a female and FE/PLB/2 was a male, both from Wrocław Zoological Garden. FE/PL/1 was a male from a zoo in the Czech Republic, and FE/PL/2 was a wild male imported to Europe in 1960 from South Africa. All the above specimens are stored in the collection of the Division of Animal Anatomy at the Department of Biostructure and Animal Physiology. For BPI/C/183, BPI/C/184, BPI/C/185, BPI/C/186, and ZA17, no collection location information was available. The only known information is that BPI/C/186 was a male. BPI/4/1351 was collected in the 2000s from the Johannesburg Zoo. BPI/C/187 was collected in Morea Estate, Klaserie District, South Africa. The specimens BPI/C/183, BPI/C/184, BPI/C/185, and BPI/C/187 were collected by James Kitching between 1945 and 1990. BPI/C/183, BPI/C/184, BPI/C/185, BPI/C/186, BPI/C/187, ZA17, and BPI/4/1351 are stored at the Evolutionary Studies Institute of the University of the Witwatersrand in Johannesburg, South Africa. Due to the lack of data concerning the head-body length and tail length of the examined African lions, reference dimensions used for the contextual interpretation of orbital measurements were taken from the literature [81]. Skulls were stored dry under standard museum conditions at the respective institutions and were inspected for damage prior to measurement (see Orbital region anatomy and morphometry).

### Anatomical analysis

The following fresh samples were collected: eyeballs, upper eyelids (UE), lower eyelids (LE), superficial gland of the third eyelid (SGTE), third eyelid (TE), and lacrimal gland (LG). The nomenclature during the anatomical description was based on *Nomina Anatomica Veterinaria* [82]. Immediately after collection, gross anatomical examinations and macroscopic measurements were performed. A complete list of ocular measurements, together with definitions, anatomical landmarks, and measurement conditions (intact vs. sectioned), is provided in Table 3. Representative images are shown in Fig. 1e and h. Measurements and abbreviations were adapted from [83]. External eyeball dimensions (AED, MaEED, MiEED, MaCD, MiCD, see Table 4) were obtained on the intact eyeball prior to sectioning. Eyeball measurements were taken externally and after sectioning the eyeball; ocular adnexa were also measured following dissection. To obtain internal ocular dimensions (e.g., AACD, LAL, VCD, TLL, TLW1, TLW2, see Table 4), each eyeball was opened using a single standardized section through the optical axis, and measurements were taken immediately after sectioning to minimize tissue deformation and chamber collapse. AACD was measured as the distance from the posterior corneal surface to the anterior lens surface along the optical axis on the freshly sectioned eyeball. Prior to fixation, the globes and selected adnexal tissues were incised to facilitate fixative penetration. After completion of macroscopic measurements, tissues were placed in 4% buffered formaldehyde for at least 72 h.

**Table 4:** Morphometry (mm) (mean ± SD) of the eyeball in clouded leopard (*n* = 1), Angola lions (male, *n* = 1 and female, *n* = 1), and Pallas’s cat (*n* = 1). Values are reported as mean ± SD from six measurements per side; the animal-level sample size was *n* = 1 per group

	AED	MaEED	MiEED	MaCD	MiCD	CAT	CPT	SLT	SET	SONT	CRW	CPL	ACD	LAL	VCD	TLL	TLW1	TLW2	OND
Clouded leopard female	25.522 ± 0.8	23.815 ± 0.6	22.993 ± 0.8	18.285 ± 0.4	17.489 ± 0.5	1.212 ± 0.08	1.95 ± 0.1	0.574 ± 0.07	0.796 ± 0.1	1.968 ± 0.1	1.179 ± 0.03	3.422 ± 0.4	3.334 ± 0.1	10.17 ± 0.07	13.27 ± 0.1	22.006 ± 0.6	12.885 ± 0.5	10.14 ± 0.5	1.535 ± 0.1
Angola lion male	32.215 ± 0.5	37.814 ± 0.5	35.741 ± 0.4	25.575 ± 0.5	22.755 ± 0.5	0.985 ± 0.2	1.394 ± 0.1	1.785 ± 0.06	1.065 ± 0.1	2.113 ± 0.09	2.928 ± 0.2	6.055 ± 0.2	6.34 ± 0.2	11.315 ± 0.05	17.99 ± 0.2	30.731 ± 0.7	20.691 ± 0.7	16.041 ± 0.7	1.845 ± 0.03
Angola lion female	27.398 ± 0.6	34.0 ± 0.7	31.429 ± 0.5	21.97 ± 0.5	21.905 ± 0.5	0.965 ± 0.09	1.333 ± 0.06	1.637 ± 0.07	0.959 ± 0.1	1.948 ± 0.1	2.812 ± 0.1	7.713 ± 0.3	5.884 ± 0.2	12.211 ± 0.1	17.665 ± 0.5	26.071 ± 0.6	19.047 ± 0.7	14.17 ± 0.6	1.54 ± 0.05
Pallas’s cat male	22.92 ± 0.5	24.861 ± 0.8	23.265 ± 0.5	16.03 ± 0.5	15.685 ± 0.5	1.346 ± 0.07	0.669 ± 0.04	0.51 ± 0.07	0.386 ± 0.04	0.953 ± 0.1	1.237 ± 0.05	4.635 ± 0.3	3.189 ± 0.04	6.517 ± 0.05	10.604 ± 0.4	18.63 ± 0.5	12.204 ± 0.5	9.012 ± 0.4	1.725 ± 0.03

*AED* Axial Eye Diameter, *MaEED* Maximum Equatorial Eye Diameter, *MiEED* Minimum Equatorial Eye Diameter, *MaCD* Maximum Corneal Diameter, *MiCD* Minimum Corneal Diameter, *CAT* Corneal Axial Thickness, *CPT* Corneal Peripheral Thickness, *SLT* Scleral Limbus Thickness, *SET* Scleral Equator Thickness, *SONT* Scleral Optic Nerve Thickness, *CRW* Ciliary Ring Width, *CPL* Ciliary Processes Length, *ACD* Anterior Chamber Depth, *LAL* Lens Axial Length, *VCD* Vitreous Chamber Depth, *TLL* Tapetum Lucidum Length, *TLW1* Tapetum Lucidum Width 1 (measured at the widest part of the tapetum lucidum), *TLW2* Tapetum Lucidum Width 2 (measured at the narrowest part of the tapetum lucidum), *OND* Optic Nerve Diameter

Morphometric measurements (six repeated measurements per parameter per side, selected from the available material) of the eyeball and selected accessory organs of the eye (length, width, thickness) were conducted bilaterally using a GRIP digital sliding caliper with an accuracy of 0.01 mm (± 0.02 mm > 100 mm) (Handy Worth, Poland). For each parameter, the same anatomical landmarks were used across specimens, and caliper measurements were performed by the same operator whenever feasible. Anatomical images were captured with a Nikon D300s camera equipped with a Tamron AF 17–50 mm F/2.8 [IF] Ø 67 lens. Because the material was collected post-mortem and subsequently dissected and fixed, the resulting measurements should be regarded as descriptive values for the examined material and not as absolute species-level reference standards.

### Orbital region anatomy and morphometry

**Fig. 2:**
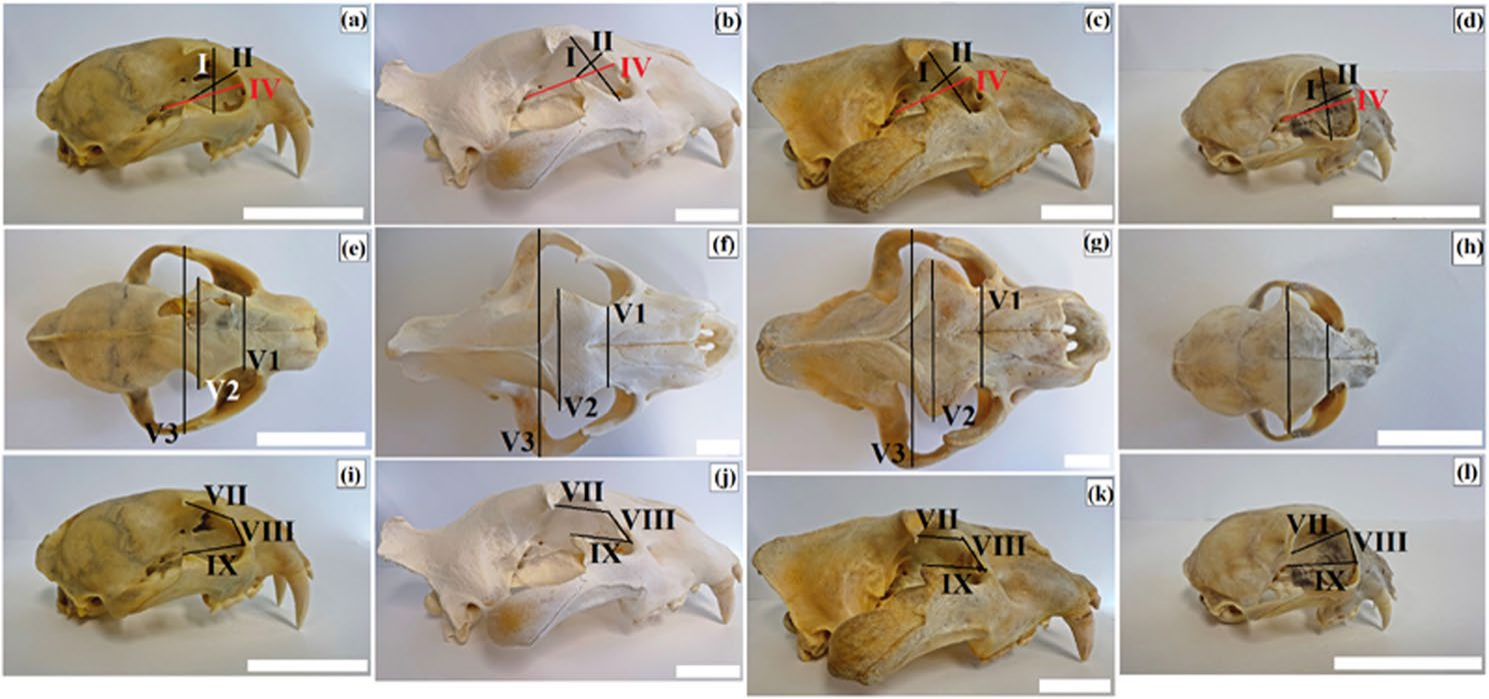
Macrographs of the orbital parameters in the clouded leopard (*Neofelis nebulosa*) (**a**, **e**, **i**), the African lions (*Panthera leo*) (**b**, **f**, **j**), the Angola lions (*Panthera leo bleyenberghi*) (**c**, **g**, **k**), and the Pallas’s cat (*Otocolobus manul* including *O. m. manul*) (**d**, **h**, **l**). I – orbital vertical length: the perpendicular distance between the supraorbital and infraorbital margins of the orbit; II – orbital horizontal width: the distance between the rostral and caudal margins of the orbital rim; IV – orbital depth: distance between the optic canal and center of the orbital rim (red line); VI1 – interorbital distance: at the rostral level (distance between the junctions of the frontolacrimal sutures on either side at the rostral margin of the orbit); VI2 – interorbital distance: at the middle level (distance between the supraorbital borders of the orbit on either side); VI3 – interorbital distance: at the caudal level (distance between the junctions of the zygomatic bone at the caudal margin of the orbit on either side); VII – frontal length: distance from the tip of the zygomatic process of the frontal bone to the frontolacrimal sutures; VIII – lacrimal length: distance from the frontolacrimal sutures to the junction between the lacrimal and zygomatic bones; IX – malar length: distance from the junction between the lacrimal and zygomatic bones to the tip of the frontal process of the zygomatic bones. Scale bars: a – l = 5 cm

The morphology of the orbital region was examined in the 14 skulls of the studied felid species and was described on the basis of domestic cat anatomy [84]. The following orbital parameters were measured (with six repeated measurements per parameter, where feasible) based on the anatomical description of the orbit of the domestic cat [85] (Fig. 2a and l). Specimens BPI/C/186, ZA17, and BPI/4/1351 were measured at the caudal level, defined as the distance between the junctions of the zygomatic bone at the caudal margin of the orbit on either side (interorbital distance), using a measuring scale. Some measurements were not determined (n.d.) due to cranial damage that precluded accurate measurement. Specifically, this applies to specimens BPI/C/185, BPI/4/1351, and BPI/C/186.

The following orbital measurements were carried out according to A. von den Driesch [85]: I, orbital vertical length; II, orbital horizontal width; III, orbital index; IV, orbital depth; V, interorbital distance (V1–V3); VI, orbital area; VII, frontal length; VIII, lacrimal length; and IX, malar length. The definitions of these measurements are given below.

I. Orbital vertical length (mm): the perpendicular distance between the supraorbital and infraorbital margins of the orbit,
II. Orbital horizontal width (mm): the distance between the rostral and caudal margins of the orbital rim (*ectorbitale – entorbitale*),
III. Orbital index (%): orbital width (B)/orbital length (A) x 100%,
IV. Orbital depth (mm): the distance between the optic canal and the center of the orbital rim,
V. Interorbital distance (mm):
  V 1. at the rostral level: the distance between the junctions of the frontolacrimal sutures on either side at the rostral margin of the orbit (the least breadth between orbits, *entorbitale – entorbitale*),
  V 2. at the middle level: the distance between the supraorbital borders of the orbit on either side (the greatest breadth between orbits, *ectorbitale – ectorbitale*),
  V 3. at the caudal level: the distance between the junctions of the zygomatic bone at the caudal margin of the orbit on either side (the greatest breadth of skull, *zygion –zygion*),
VI. Orbital area (mm^2^): π x (½ A x ½ B),
VII. Frontal length (mm): the distance from the tip of the zygomatic process of the frontal bone to the frontolacrimal sutures,
VIII. Lacrimal length (mm): the distance from the frontolacrimal sutures to the junction between the lacrimal and zygomatic bones,
IX. Malar length (mm): the distance from the junction between the lacrimal and zygomatic bones to the tip of the frontal process of the zygomatic bone.

### CT scanner

Computed tomography of the skull was performed using a 64-slice, 128-slice Siemens SOMATOM go.TOP CT scanner (Siemens Healthcare GmbH (Siemens Healthineers), Erlangen, Germany) in the Department and Clinic of Surgery, Wrocław University of Environmental and Life Sciences in Wrocław. Scanning was performed in the long axis of the skull, first in the cranial direction and then in the caudal direction. Each of the skulls was scanned separately. The following exposure parameters were used during the CT scan: 120 kV and 60 mAs. Cross-sectional images were obtained using a bone filter with a slice thickness of 0.6 mm. Images were post-processed using the syngo.via software (Siemens Healthineers). In addition, multiplanar (sagittal, dorsal, and transverse sections) and three-dimensional reconstructions were generated. CT imaging facilitated visualization of selected bony orbital structures and supported morphometric assessment.

### Light microscopic studies

The examined samples (eyeballs and selected accessory organs of the eye) were placed in 4% buffered formaldehyde for at least 72 h and then rinsed in running water for 24 h. Subsequently, the material was processed using a vacuum tissue processor (ETP RVG3, Intelsint, Italy) and embedded in paraffin wax. Section  (4 μm thick) were cut using a sliding microtome (Slide 2003, Pfm A.G., Germany). Half of the slides were stained using Mayer’s hematoxylin and eosin; Heidenhain’s Azan trichrome (Bio-Opitca Milano S.p.A., Milan, Italy; cat. no. 04-001802) (collagen fibers and mucus – blue color); Movat pentachrome (modified Russell-Movat) (BioGnost Ltd., Zagreb, Croatia; cat. no. MOV-100T) for acid mucopolysaccharides and glycosaminoglycans (GAGs) – blue to green; Mallory trichrome with aniline blue (Bio-Optica Milano S.p.A., Milan, Italy; cat. no. 04-020802) (collagen fibers and mucus – blue color; keratin and muscle fibers – red color); Masson-Goldner trichrome (Bio-Opitca Milano S.p.A., Milan, Italy; cat. no. 04-011802) (collagen fibers and mucus – green color; keratin and muscle fibers – red color); and Masson-Fontana (BioGnost Ltd., Zagreb, Croatia; cat. no. MF-100T) to visualize melanin granules (black) [86–88].

The following measurements (µm) were taken [89]:

I. Anterior corneal epithelium thickness (ACET),
II. Proper substance of the cornea thickness (PSCT),
III. Posterior limiting membrane thickness (PLMT),
IV. Tapetum lucidum cells diameter (*TL*CD),
V. Tapetum lucidum thickness (*TL*T),
VI. Choroid thickness (ChT).

Morphometric measurements (six repeated measurements per structure) were performed using a Zeiss Axio Scope A1 light microscope (Carl Zeiss, Jena, Germany) with Axio Vision Release 4.8.2 SP 2 software. Repeated measurements were not treated as independent biological replicates, and no inferential statistical tests were performed.

The remaining slides were stained with periodic acid-Schiff (PAS) (Bio-Optica Milano S.p.A., Milan, Italy; cat. no. 04-130802); alcian blue pH 1.0 (AB pH 1.0) (Bio-Optica Milano S.p.A., Milan, Italy; cat. no. 04-166802); alcian blue pH 2.5 (AB pH 2.5) (Bio-Optica Milano S.p.A., Milan, Italy; cat. no. 04-160802); alcian blue pH 2.5/periodic acid-Schiff (AB pH 2.5/PAS) (Bio-Optica Milano S.p.A., Milan, Italy; cat. no. 04-163802); and colloidal iron (CI) (Bio-Optica Milano S.p.A., Milan, Italy; cat. no. 04-180809) methods [90]. Histochemical studies aimed to evaluate the composition of secretory units of goblet cells and orbital glands, following methods described by Spicer and Henson [90]. The results were interpreted as follows: (–) indicates a negative reaction, (–/+) and (+) indicate a weak positive reaction, (++) and (++/+++) indicate a moderate positive reaction, and (+++) indicates a strong positive reaction. Furthermore, the following histochemical stains were used to evaluate the composition of glandular secretion: PAS, AB pH 1.0, AB pH 2.5, AB pH 2.5/PAS, and CI:

- PAS characterizes glycans, glycoconjugates, and neutral glycoproteins,
- AB pH 1.0 visualizes strongly sulfated mucosubstances,
- AB pH 2.5 identifies sialylated acid glycosaminoglycans,
- AB pH 2.5/PAS identifies sulfated and carboxylated acid mucopolysaccharides, sulfated and carboxylated sialomucins (glycoproteins) (blue color), and neutral mucins (magenta color),
- CI detects the presence of sulfated acid mucosubstances (SAM) and carboxylated acid mucosubstances (CAM).

All histological and histochemical slides were examined using a Zeiss Axio Scope A1 light microscope (Carl Zeiss, Jena, Germany) with Axio Vision Release 4.8.2 SP 2 software. The nomenclature during histological descriptions followed *Nomina Histologica Veterinaria* [91, 92].

### Statistical analysis

The limited number of individuals allowed only descriptive summary of the measurements (mean and standard deviation), which were calculated to summarize repeated measurements. These repeated measurements were used to characterize the examined material only and were not treated as independent biological replicates; therefore, no inferential statistical tests were performed.

## Results

### The eyeball morphology

The axial eye diameter (AED), maximum equatorial eye diameter (MaEED), and minimum equatorial eye diameter (MiEED) values are reported in Table 4. Among the examined individuals, the male Angola lion had the largest AED and MaEED, whereas the male Pallas’s cat had the smallest AED. Comparison of MaEED and MiEED provided a descriptive assessment of eyeball shape in the equatorial plane. The largest differences between these values were recorded in the Angola lions, the smallest in the clouded leopard, and an intermediate difference in the Pallas’s cat (Table 4).

**Fig. 3:**
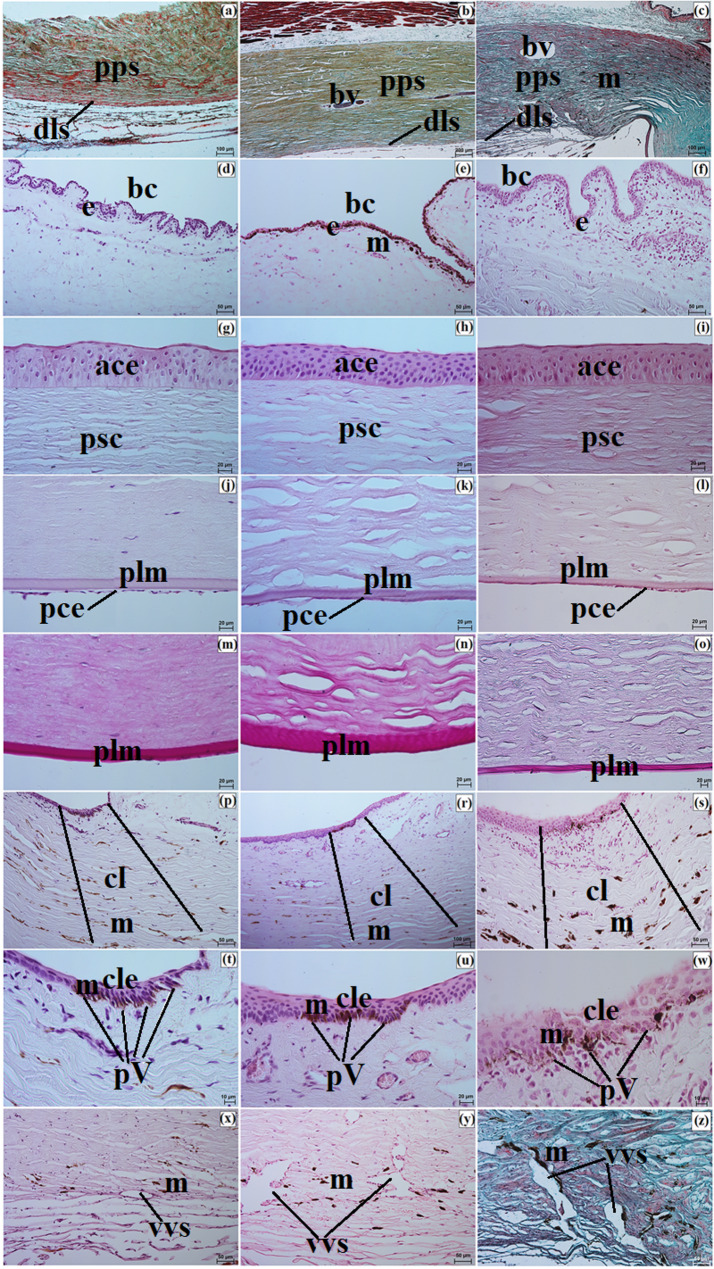
Micrographs of the sclera (**a-c**), bulbar conjunctiva (**d-f**), cornea (**g-o**), palisades of Vogt (**p-w**), and the venous sinus of the sclera (**x-z**) in the clouded leopard (*Neofelis nebulosa*) (**a**, **d**, **g**, **j**, **m**, **p**, **t**, **x**), the Angola lions (*Panthera leo bleyenberghi*) (**b**, **e**, **h**, **k**, **n**, **r**, **u**, **y**), and the Pallas’s cat (*Otocolobus manul manul*) (**c**, **f**, **i**, **l**, **o**, **s**, **w**, **z**). ace – anterior corneal epithelium, bc – bulbar conjunctiva, bv – blood vessels, cl – corneal limbus, cle – corneal limbus epithelium, dls – dark lamina of the sclera, e – epithelium, m – melanocytes, pce – posterior corneal epithelium, plm – posterior limiting membrane, psc – proper substance of the cornea, pss – proper substance of the sclera, pV – palisades of Vogt, vvs – venous vessels of the sclera. a – c, z = Movat pentachrome (modified Russell Movat) stain; d – l, p – y = Mayer’s H&E stain; m – o = PAS stain. Scale bars: b = 200 μm; a, c, r = 100 μm; d – f, p, s, x – z = 50 μm; g – o, u = 20 μm; t, w = 10 μm. Magnifications: 5x – b; 10x – a, c, v; 20x – d, e, f, p, s, u, x, y, z; 40x – g, h, i, j, k, l, m, n, o; 63x – t, w

The sclera consisted of three layers: the episcleral lamina, the proper substance, and the dark lamina. The episcleral lamina was composed of loose fibrous connective tissue, whereas the proper substance contained predominantly collagen fibers with faint elastic fibers arranged in intersecting bundles. In the Pallas’s cat, this layer also contained numerous blood vessels and melanin granules, whereas in the Angola lions and the clouded leopard, blood vessels were sparse and melanin granules were not observed. The dark lamina was thinner in the Angola lions and thicker in the clouded leopard and the Pallas’s cat (Fig. 3a and c).

The bulbar conjunctiva covered the anterior surface of the eyeball over a distance of 2.367 ± 0.5 mm in the clouded leopard, 5.54 ± 1.6 mm in the male Angola lion, 5.166 ± 1.1 mm in the female Angola lion, and 3.1 ± 0.4 mm in the Pallas’s cat. It was lined by a nonkeratinized stratified squamous epithelium with 3 to 4 layers in the clouded leopard, 6 to 9 layers in the Angola lions, and 7 to 8 layers in the Pallas’s cat. Melanin granules were present in the epithelium of the Angola lions. The underlying connective tissue was loose and fibrous (Fig. 3d and f). Samples were collected from the dorsal bulbar conjunctiva, approximately 5 mm posterior to the corneal limbus, from a single site in each individual.

Maximum corneal diameter (MaCD) and minimum corneal diameter (MiCD) are presented in Table 4. The male Angola lion had the largest MaCD, whereas the male Pallas’s cat had the smallest cornea. The difference between MaCD and MiCD indicated that the corneal outline varied among the examined individuals, with the greatest difference in the male Angola lion and the smallest in the female Angola lion (Table 4). The cornea consisted of four layers, as no anterior limiting membrane (Bowman membrane) was observed in the examined material. The anterior corneal epithelium was a nonkeratinized stratified squamous epithelium with 7 to 8 layers in the clouded leopard, 11 to 12 layers in the Angola lions, and 6 to 8 layers in the Pallas’s cat (Fig. 3g and i). It consisted of superficial squamous cells, intermediate prickle cells, and basal cuboidal cells. The proper substance of the cornea was composed of dense fibrous connective tissue with collagen fibers and numerous corneocytes. The posterior limiting membrane (Descemet’s membrane) showed a strong (+++) PAS-positive reaction, and the posterior corneal epithelium consisted of a single layer of flat cells (Fig. 3j and o).

The measurement results for anterior corneal epithelium thickness (ACET), proper substance of the cornea thickness (PSCT), and posterior limiting membrane thickness (PLMT) are presented in Table 5. Among the examined individuals, the female clouded leopard had the greatest ACET, the male Angola lion had the greatest PSCT, and PLMT was greatest in the Angola lions, intermediate in the clouded leopard, and lowest in the Pallas’s cat (Table 5).

**Table 5:** Morphometry (µm) (mean ± SD) of the histological structures of the eyeball in clouded leopard (*n* = 1), Angola lions (male, *n* = 1 and female, *n* = 1), and Pallas’s cat (*n* = 1). Values are reported as mean ± SD from six measurements per side in each individual; the animal-level sample size was *n* = 1 per group

	ACET	PSCT	PLMT	TLCD	TLT	ChT
Clouded leopardfemale	60.781± 1.8	295.53± 7.6	11.764± 0.7	53.161± 4.6	96.753± 2.9	106.103± 27.5
Angola lion male	51.54 ± 2.3	307.141 ± 4.4	15.211 ± 1.1	70.596 ± 13.7	145.601 ± 10.9	201.237 ± 29.5
Angola lion Female	51.356 ± 1.3	301.355 ± 5.9	13.743 ± 0.3	64.133 ± 7.0	133.154 ± 6.2	192.316 ± 31.5
Pallas’s Cat male	56.746 ± 9.5	289.117 ± 8.2	8.859 ± 0.3	37.330 ± 6.5	74.736 ± 2.6	107.452 ± 12.0

*ACET* Anterior Corneal Epithelium Thickness, *PSCT* Proper Substance of the Cornea Thickness, *PLMT* Posterior Limiting Membrane Thickness, *TLCD* Tapetum Lucidum Cells Diameter, *TLT* Tapetum Lucidum Thickness, *ChT* Choroid Thickness

**Fig. 4:**
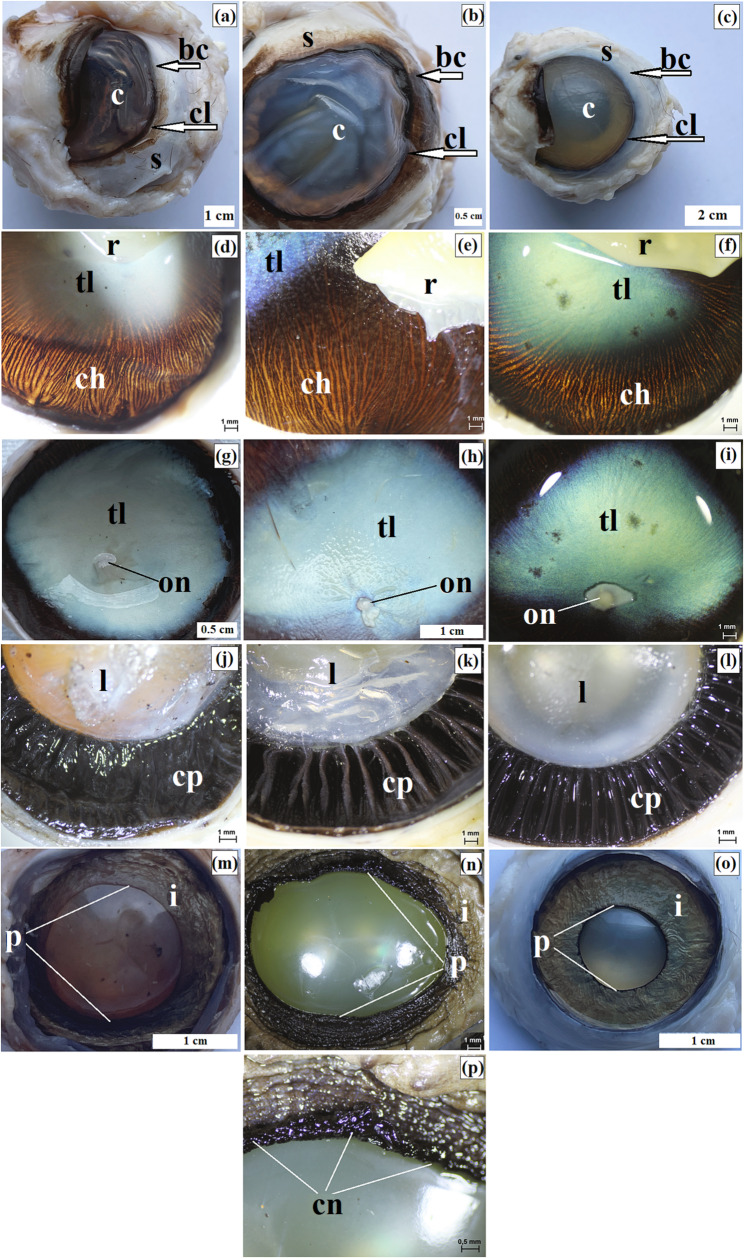
Macrographs of the eyeball in the clouded leopard (*Neofelis nebulosa*) (**a**, **d**, **g**, **j**, **m**), the Angola lions (*Panthera leo bleyenberghi*) (**b**, **e**, **h**, **k**, **n**, **p**), and the Pallas’s cat (*Otocolobus manul manul*) (**c**, **f**, **i**, **l**, **o**). bc – bulbar conjunctiva, c – cornea, ch – choroid, cl – corneal limbus, cn – corpora nigra (iridic granule), cp. – ciliary processes, i – iris, l – lens, on – optic nerve, p – pupil, r – retina, s – sclera, tl – tapetum lucidum. Scale bars: c = 2 cm; a, h, m, o = 1 cm; b, g, p = 0.5 cm; d – f, i – l, n = 1 mm; p = 0.5 mm

At the corneal limbus, Vogt palisades were visible. Histologically, the limbus contained blood and lymph vessels as well as melanin granules, which were more numerous in the clouded leopard and Pallas’s cat than in the Angola lions (Fig. 3p and s). The limbal epithelium was multilayered in all examined individuals, and a slight accumulation of melanocytes was present in the basal cells (Fig. 3t and w). The iridocorneal angle contained scleral venous vessels lined with flat endothelial cells, and the scleral venous sinus was bordered by the pectinate ligament (Fig. 3x and z). Macroscopically, the corneal limbus was strongly pigmented in the clouded leopard and Angola lions and slightly pigmented in the Pallas’s cat (Fig. 4a and c).

Corneal axial thickness (CAT), corneal peripheral thickness (CPT), scleral limbus thickness (SLT), scleral equator thickness (SET), and scleral optic nerve thickness (SONT) are summarized in Table 4. CAT was greatest in the Pallas’s cat, CPT in the clouded leopard, and SLT and SET in the Angola lions. SONT was greatest in the Angola lions and the clouded leopard and lowest in the Pallas’s cat (Table 4).

Macroscopically, the tapetum lucidum appeared as an opalescent crescent, milky blue in the clouded leopard and Angola lions and blue-green in the Pallas’s cat (Fig. 4d and i). Tapetum lucidum length (TLL), width at the widest point (TLW1), and width at the narrowest point (TLW2) are presented in Table 4. The largest TLL, TLW1, and TLW2 values were observed in the Angola lions. In these two individuals, the tapetum lucidum had the greatest difference between TLW1 and TLW2, indicating the most elongated outline among the examined animals.

The corpus ciliare formed the thickest part of the uveal tract and extended from the dentate line to the ciliary margin of the iris. Measurements of ciliary ring width (CRW) and ciliary process length (CPL) are presented in Table 4; Fig. 4j and l. The ciliary processes contained supporting tissue rich in fenestrated capillaries. Their number ranged from 83 to 84 to 88–89 in the clouded leopard, from 82 to 86 to 94–96 in the Angola lions, and from 83 to 84 to 86–87 in the Pallas’s cat. The ciliary body was lined by a double layer of cuboidal epithelium and contained a well-developed ciliary muscle.

The iris was light brown in the clouded leopard and Angola lions and yellowish-blue in the Pallas’s cat (Fig. 4m and o). Histologically, it consisted of an anterior epithelium, an outer limiting layer with collagen fibers, fibrocytes, and melanin granules, and a stroma containing blood vessels, delicate collagen fibers, and nerves. The pupil was round at rest in the clouded leopard and Pallas’s cat and horizontally ovoid in the Angola lions (Fig. 4m and o). An iridic granule (corpora nigra) was observed only in the Angola lions (Fig. 4p).

**Fig. 5:**
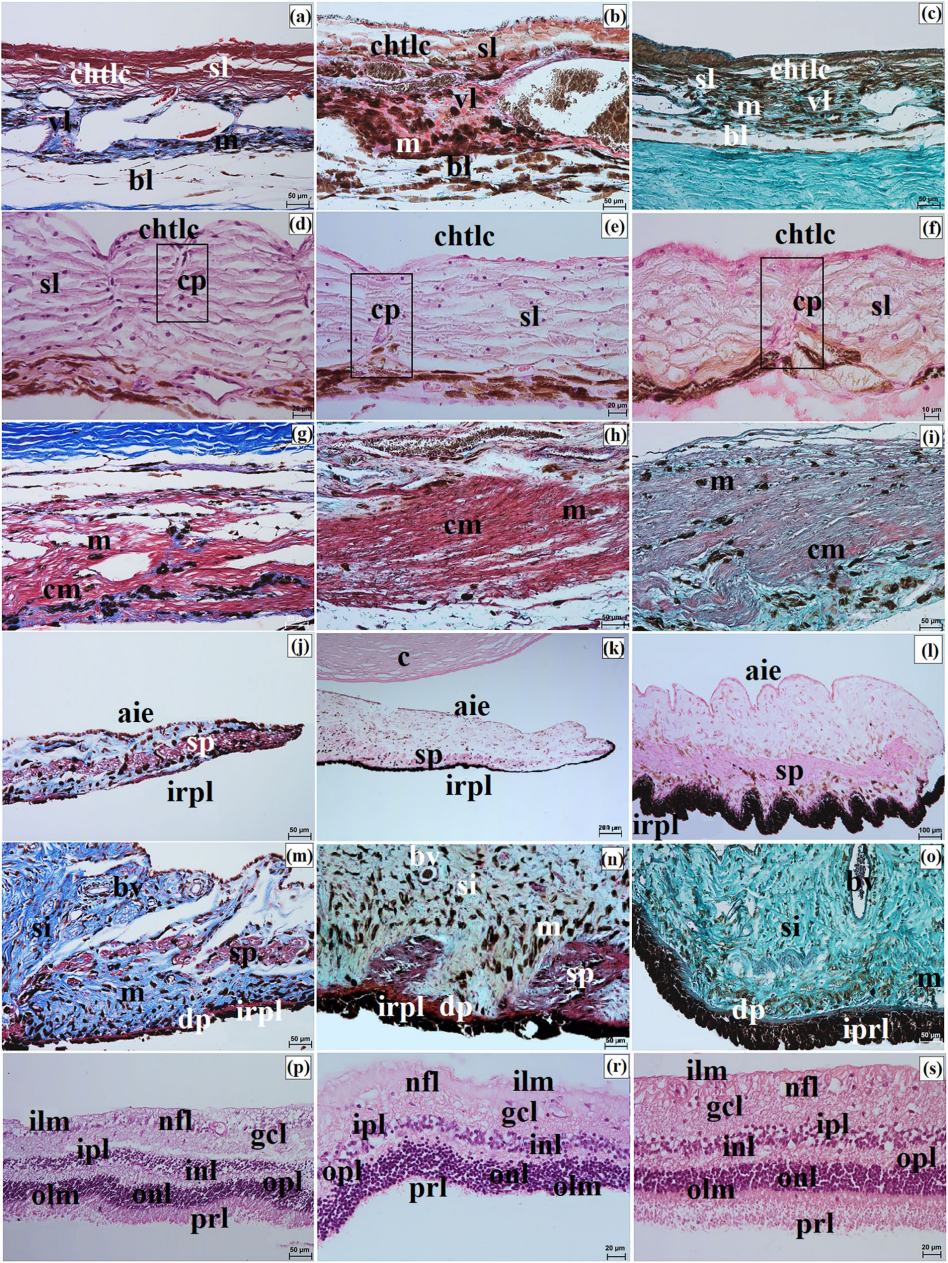
Micrographs of the choroid (**a-c**), *tapetum lucidum* (**g-f**), ciliary muscle (**g-i**), iris (**j-o**), and the retina (**p-s**) in the clouded leopard (*Neofelis nebulosa*) (**a**, **d**, **g**, **j**, **m**, **p**), the Angola lions (*Panthera leo bleyenberghi*) (**b**, **e**, **h**, **k**, **n**, **r**), and the Pallas’s cat (*Otocolobus manul manul*) (**c**, **f**, **i**, **l**, **o**, **s**). aie – anterior iris epithelium, bl – basal layer, bv – blood vessels, c – cornea, chtlc – choroidal tapetum lucidum cellulosum, cm – ciliary muscle, cp. – capillary, dp – dilator of pupil, gcl – ganglion cell layer, ilm – internal limiting membrane, inl – inner nuclear layer, ipl – inner plexiform layer, irpl – iris pigmentosum layer, m – melanocytes, nfl – nerve fiber layer, olm – outer limiting membrane, onl – outer nuclear layer, opl – outer plexiform layer, prl – photoreceptor layer (rods and cones), si – stroma of iris, sl – suprachoroid layer, sp – sphincter of pupil, vl – vascular layer. a, g, j, m = Mallory trichrome with aniline blue stain; b, h, n = Movat pentachrome (modified Russell Movat) stain; c, i, o = Masson-Goldner trichrome stain; d – f, k, l = Mayer’s H&E stain. Scale bars: k = 200 μm; l = 100 μm; a – c, g – j, m – p = 50 μm; d, e, r, s = 20 μm; f = 10 μm. Magnifications: 5x – k; 10x – l; 20x – a, b, c, g, h, i, j, m, n, o, p; 40x – d, e, r, s; 63x – f

The choroid consisted of five layers. In the suprachoroidal layer, numerous melanin granules were present in the Angola lions and the Pallas’s cat, whereas they were not observed in the clouded leopard. The vascular layer contained large arterial and venous vessels together with smaller vessels embedded in loose connective tissue with melanocytes (Fig. 5a and c). The tapetum lucidum cellulosum was composed of large polygonal cells arranged in multiple layers, with capillary vessels present between them (Fig. 5d and f). The greatest tapetum lucidum cell diameter (TLCD) and tapetum lucidum thickness (TLT) were recorded in the male Angola lion, whereas the smallest values were observed in the Pallas’s cat. Choroid thickness (ChT) was greatest in the Angola lions and lowest in the clouded leopard and Pallas’s cat (Table 5). The lamina of capillary vessels and Bruch’s membrane were also identified (Fig. 5a and c).

Numerous clusters of melanocytes were observed between the ciliary muscle fibers (Fig. 5g and i). Anterior chamber depth (ACD) was greater in both Angola lions than in the clouded leopard and Pallas’s cat; the greatest value was recorded in the male Angola lion and the smallest in the Pallas’s cat (Table 4). The iris stroma formed well-developed sphincter and dilator pupillae muscles (Fig. 5j and o). On the posterior surface of the iris, a retinal pigment epithelium composed of two layers of melanin-containing cells was present.

The nervous part of the retina consisted of 10 layers arranged in the typical sequence seen in terrestrial mammals (Fig. 5p and s). The optic disc was oval and had a milky white fundus with delicate blood vessels visible (Fig. 4g and j). The lamina cribrosa of the sclera was present. The lens was biconvex and round. Axial lens length (LAL) was greater in both Angola lions than in the clouded leopard and Pallas’s cat, whereas the shortest value was recorded in the Pallas’s cat (Table 4). Vitreous chamber depth (VCD) was greatest in the Angola lions, intermediate in the clouded leopard, and smallest in the Pallas’s cat. Optic nerve diameter (OND) was greater in the male Angola lion and the Pallas’s cat than in the female Angola lion and the clouded leopard (Table 4).

### The upper and lower eyelids morphology

The measurement results of the upper and lower eyelids (UE and LE) in the examined Felidae are presented in Table 6. Among the examined individuals, the Angola lions had the largest eyelid dimensions, whereas the Pallas’s cat had the smallest. The clouded leopard showed intermediate values. Differences were also noted between the male and female Angola lions, including eyelid length, width, and thickness. Variability across the six measurements per side is summarized in Table 6.

**Table 6:** Morphometry (mm) (mean ± SD) of selected accessory organs of the eye in clouded leopard (*n* = 1), Angola lions (male, *n* = 1 and female, *n* = 1), and Pallas’s cat (*n* = 1)

	Upper and lower eyelids	Upper eyelid		Lower eyelid		Superficial gland of the third eyelid			Upper and lower branch of the third eyelid	Crossbar of the third eyelid	Lacrimal gland		
	length	width	thickness	width	thickness	length	width	thickness	length	length	length	width	thickness
Clouded leopard female	24.844 ± 0.4	10.0625 ± 0.3	5.012 ± 0.3	7.165 ± 0.6	6.809 ± 1.0	14.756 ± 0.8	14.297 ± 0.7	6.609 ± 0.5	14.632 ± 0.5	13.81 ± 1.11	11.155 ± 0.4	6.815 ± 0.3	4.65 ± 0.3
Angola lion male	15.979 ± 1.1	25.237 ± 0.8	7.105 ± 0.5	17.395 ± 0.8	7.92 ± 0.7	26.124 ± 1.0	22.188 ± 0.7	5.991 ± 0.3	23.29 ± 0.6	28.965 ± 0.5	17.997 ± 0.1	13.705 ± 0.3	6.092 ± 0.3
Angola lion female	43.248 ± 1.1	21.77 ± 1.0	7.295 ± 0.6	12.996 ± 0.9	7.184 ± 0.7	25.126 ± 0.7	21.56 ± 0.7	5.87 ± 0.2	23.217 ± 0.5	28.949 ± 1.0	18.215 ± 0.7	12.778 ± 0.3	5.698 ± 0.4
Pallas’s cat male	22.116 ± 1.3	14.289 ± 0.8	2.88 ± 0.4	9.149 ± 0.7	2.36 ± 0.6	9.844 ± 0.3	9.672 ± 0.5	2.997 ± 0.2	12.39 ± 0.7	10.794 ± 0.4	12.12 ± 0.3	6.586 ± 0.3	2.774 ± 0.3

Values are reported as mean ± SD from six measurements per side in each individual; the animal-level sample size was *n* = 1 per group

**Fig. 6:**
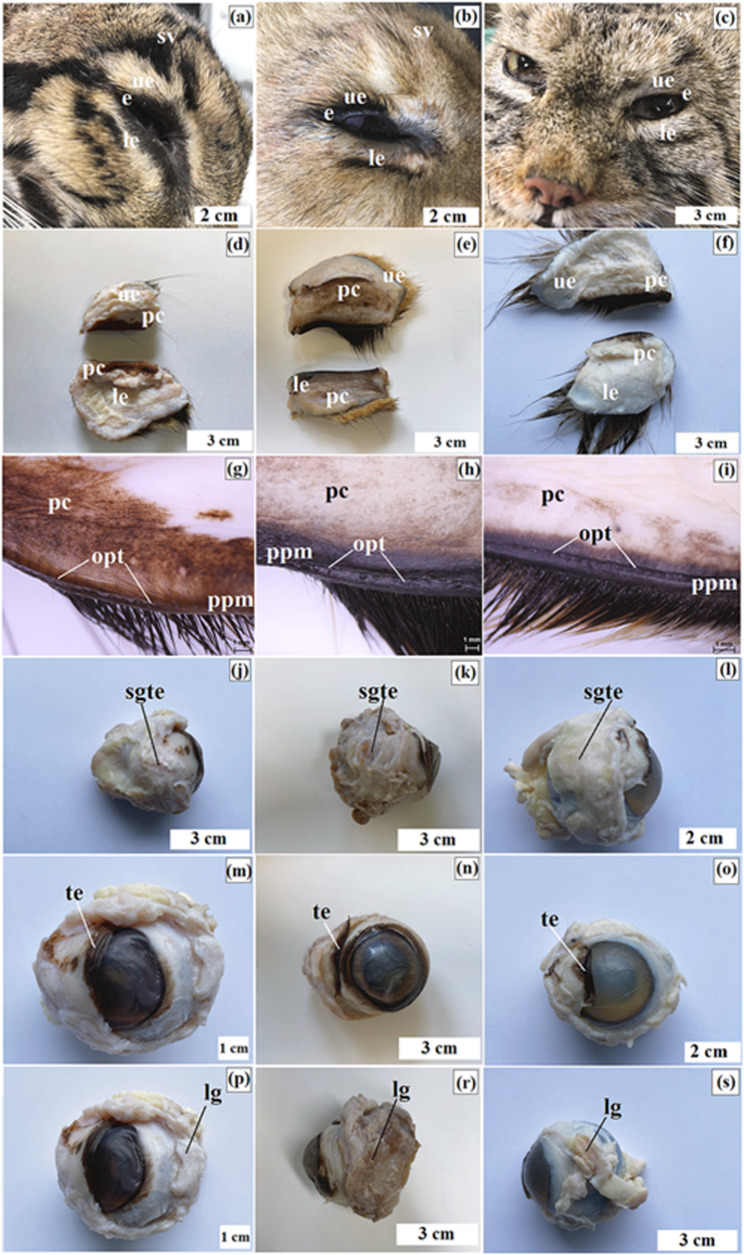
Macrographs of the upper and lower eyelids (**a-i**), the superficial gland of the third eyelid (**j-l**), the third eyelid (**m-o**), and the lacrimal gland (**p-s**) in the clouded leopard (*Neofelis nebulosa*) (**a**, **d**, **g**, **j**, **m**, **p**), the Angola lions (*Panthera leo bleyenberghi*) (**b**, **e**, **h**, **k**, **n**, **r**), and the Pallas’s cat (*Otocolobus manul manul*) (**c**, **f**, **i**, **l**, **o**, **s**). e – eyelashes, le – lower eyelid, lg – lacrimal gland, opt – opening of the tarsal gland, pc – palpebral conjunctiva, ppm – posterior palpebral margin, sgte – superficial gland of the third eyelid, te – third eyelid, up – upper eyelid, sv – supraorbital vibrissae. Scale bars: c – f, j, k, n, r, s = 3 cm; a, b, l, o = 2 cm; m, p = 1 cm; g – i = 1 mm

The brown-black eyelashes were present only on the upper eyelid and were arranged in 2–3 rows in all examined individuals (Fig. 6a and c). The supraorbital vibrissae, ranging in number from 5 to 6 to 8–10, were dark and light in the clouded leopard and Angola lions, whereas in the Pallas’s cat they were light only (Fig. 6a and c). The palpebral conjunctiva in the clouded leopard had a brown coloration and covered most of both eyelids. In the Angola lions and the Pallas’s cat, the palpebral conjunctiva near the anterior palpebral margin of both eyelids was strongly pigmented (dark brown to almost black) (Fig. 6d and i). Toward the superior and inferior conjunctival fornices, pigmentation became less intense; however, in the Angola lions it extended across the entire palpebral conjunctiva, whereas in the Pallas’s cat it was limited to approximately half of its extent (Fig. 6e, f, h and i).

**Fig. 7:**
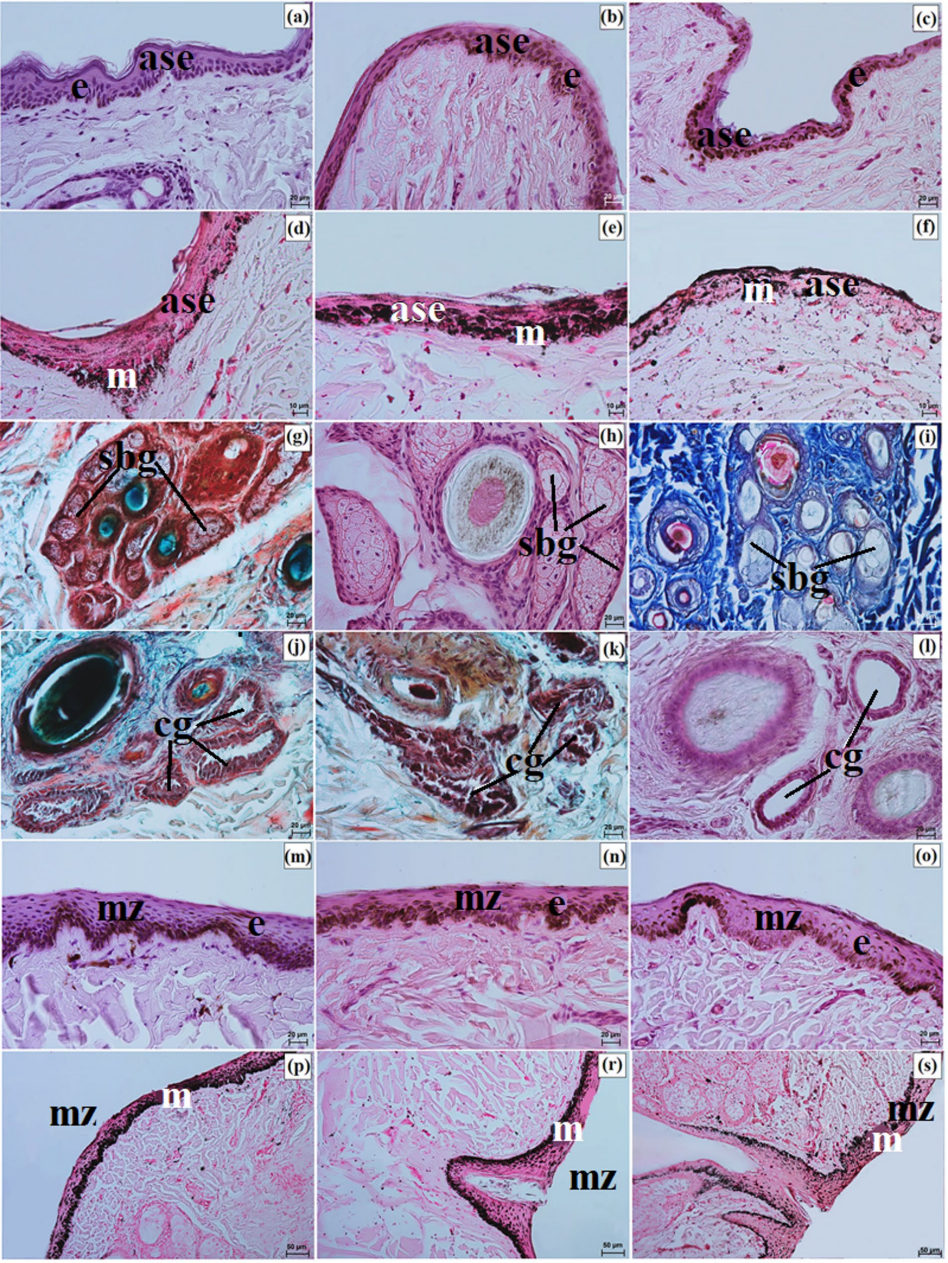
Micrographs of the upper and lower eyelids in the clouded leopard (*Neofelis nebulosa*) (**a**, **d**, **g**, **j**, **m**, **p**), the Angola lions (*Panthera leo bleyenberghi*) (**b**, **e**, **h**, **k**, **n**, **r**), and the Pallas’s cat (*Otocolobus manul manul*) (**c**, **f**, **i**, **l**, **o**, **s**). ase – anterior surface of the eyelid, cg – ciliary glands, e – epithelium, m – melanocytes, mz – marginal zone, sbg – sebaceous glands. a – c, h, l, m – o = Mayer’s H&E stain; d – f, p – s = Fontana-Masson stain; g, j, k = Movat pentachrome (modified Russell Movat) stain; i = Mallory trichrome with aniline blue stain. Scale bars: p – s = 50 μm; a – c, g – o = 20 μm; d – f = 10 μm. Magnifications: 20x – p, r, s; 40x – a, b, c, g, h, I, j, k, l, m, n, o; 63x – d, e, f

The anterior surface of the eyelids was covered by keratinized stratified squamous epithelium, with 3 to 5 layers in the Pallas’s cat, 8 to 10 in the clouded leopard, and 6 to 9 in the Angola lions (Fig. 7a and c). The stratum basale contained numerous melanocytes in the clouded leopard and Angola lions, whereas in the Pallas’s cat they were weakly marked (Fig. 7d and f). The eyelash follicles were associated with well-developed simple branched sebaceous glands and ciliary glands (Fig. 7g and l). The marginal zone was covered by non-keratinized stratified columnar epithelium, with 10 to 15 layers in the clouded leopard, 10 to 12 in the Angola lions, and 4 to 10 in the Pallas’s cat (Fig. 7m and o). In all examined individuals, this zone was strongly pigmented because of abundant melanin granules (Fig. 7p and s).

**Fig. 8:**
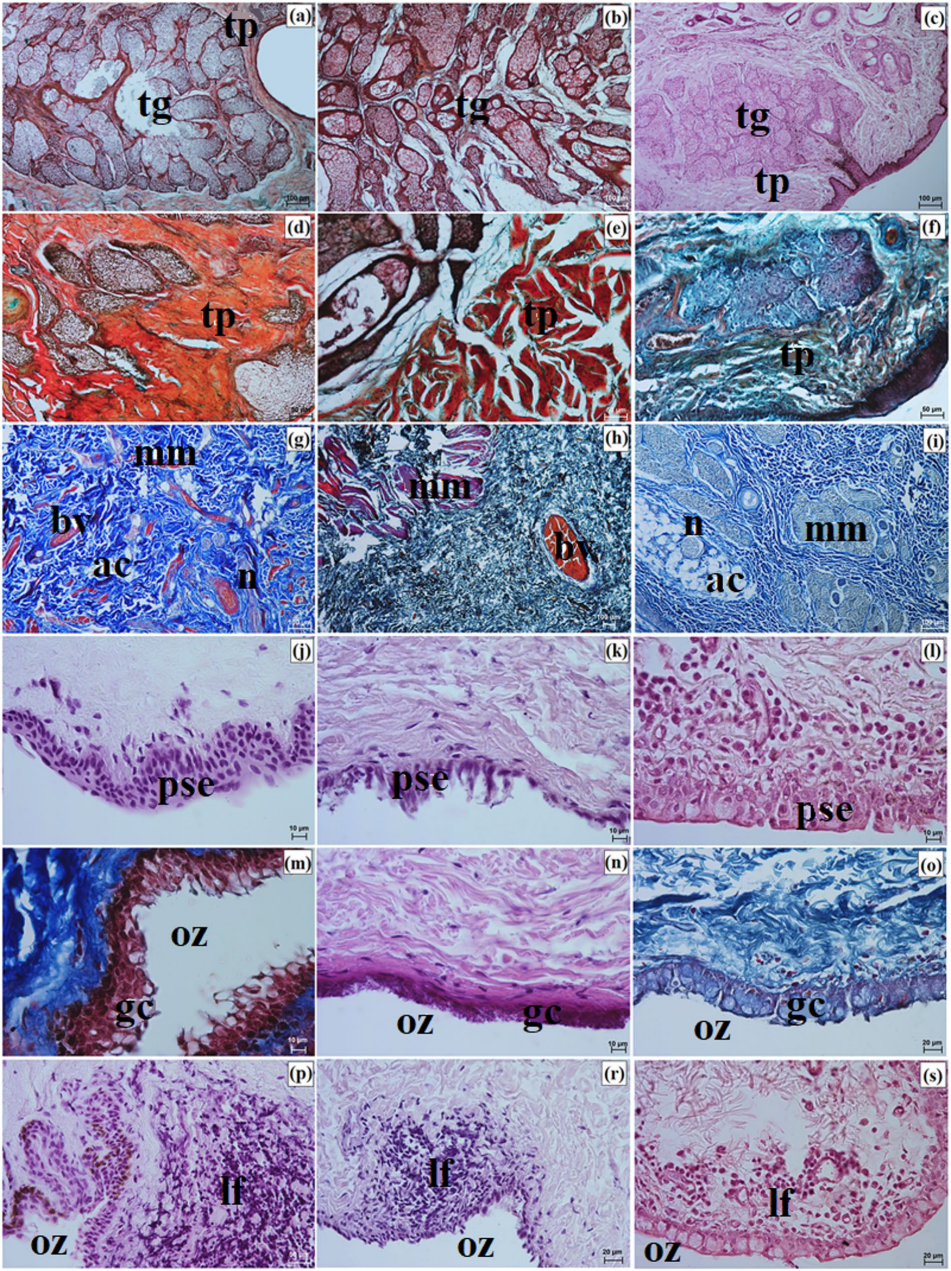
Micrographs of the upper and lower eyelids in the clouded leopard (*Neofelis nebulosa*) (**a, d, g, j, m, p**), the Angola lions (*Panthera leo bleyenberghi*) (**b, e, h, k, n, r**), and the Pallas’s cat (*Otocolobus manul manul*) (**c, f, i, l, o, s**). ac – adipose cells, bv – blood vessels, lf – lymphoid follicle, gc – goblet cells, mm – muscles, oz – orbital zone, pse – posterior surface of the eyelid, tg – tarsal glands, tp – tarsal plate. a, b, d – f = Movat pentachrome (modified Russell Movat) stain; c, j – l, n, p – s = Mayer’s H&E stain; g – i, m, n = Mallory trichrome with aniline blue stain. Scale bars: a – c, g – i = 100 μm; d – f, p = 50 μm; o, r, s = 20 μm, j – n = 10 μm. Magnifications: 10x – a, b, c, g, h, i; 20x – d. e. f. p; 40x – o, r, s; 63x – j, k, l, m, n

Well-developed tarsal glands were observed in the eyelids (Fig. 8a and c). Their number in UE and LE ranged from 26 to 29 in the clouded leopard, 35 to 39 in the Angola lions, and 31 to 33 in the Pallas’s cat (Fig. 6g and i). The tarsal plate was well developed and composed of dense fibrous connective tissue with a regular arrangement (Fig. 8a and f). The eyelid stroma in all examined felids consisted of irregularly arranged dense fibrous connective tissue containing elastic, collagen, and reticular fibers, as well as numerous blood vessels, nerves, and muscle bundles (Fig. 8g and i). The posterior palpebral surface was covered by stratified columnar epithelium (Fig. 8j and l). In the orbital zone, the conjunctiva was lined by stratified cuboidal epithelium, with 6 to 8 layers in the clouded leopard, 4 to 6 in the Angola lions, and 5 to 6 in the Pallas’s cat. This zone contained 5 to 7 conjunctival folds with numerous goblet cells (Fig. 8m and o).

**Fig. 9:**
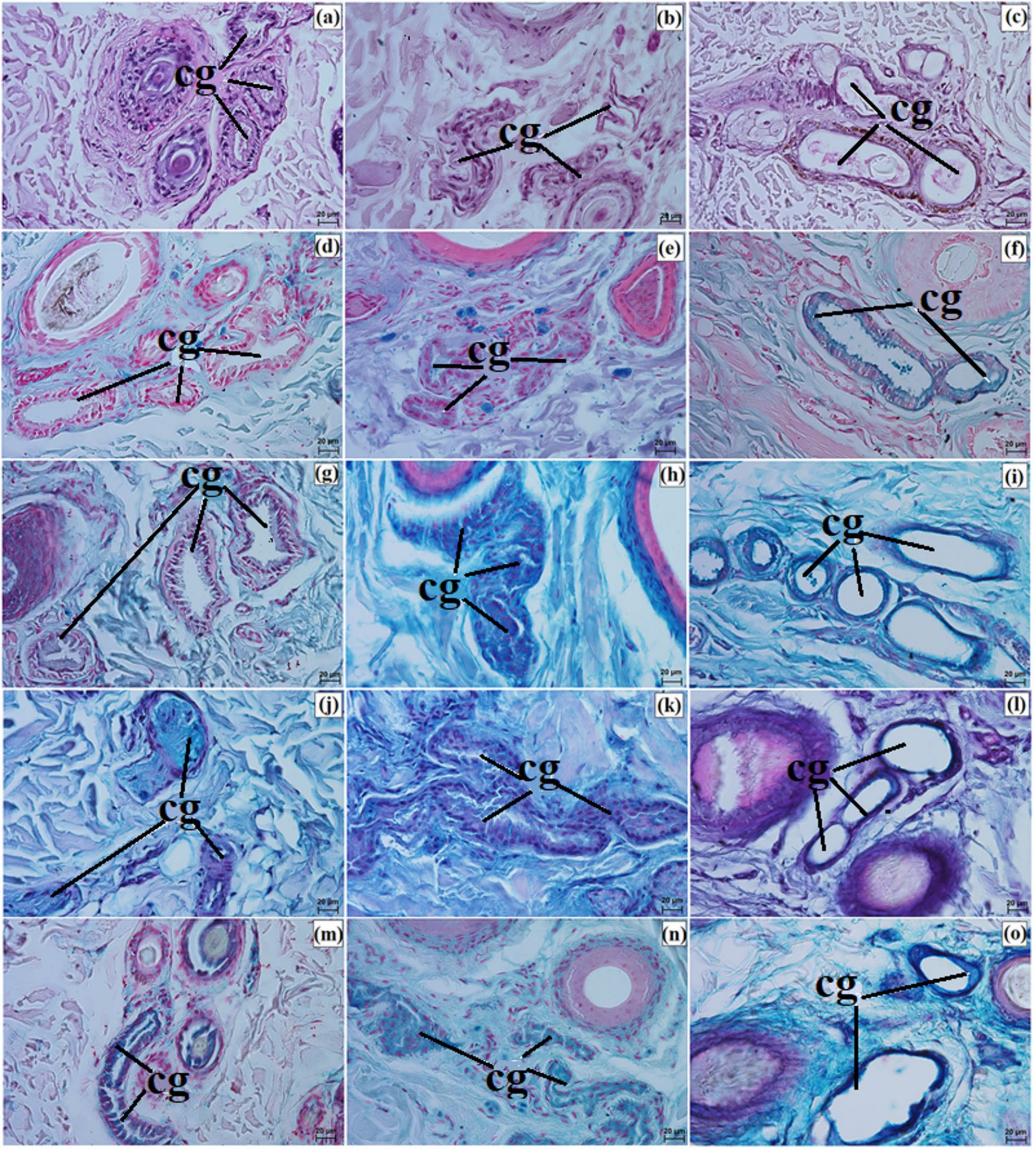
Micrographs of the histochemistry of ciliary glands (cg) in the eyelids of the clouded leopard (*Neofelis nebulosa*) (**a**, **d**, **g**, **j**, **m**), the Angola lions (*Panthera leo bleyenberghi*) (**b**, **e**, **h**, **k**, **n**), and the Pallas’s cat (*Otocolobus manul manul*) (**c**, **f**, **i**, **l**, **o**). a – c = PAS stain; d – f = AB pH 1.0 stain; g – i = AB pH 2.5 stain; j – l = AB pH 2.5/PAS stain; m – o = CI stain. Scale bars: a – o = 20 μm. Magnifications: 40x – a, b, c, d, e, f, g, h, i, j, k, l, m, n, o

**Fig. 10:**
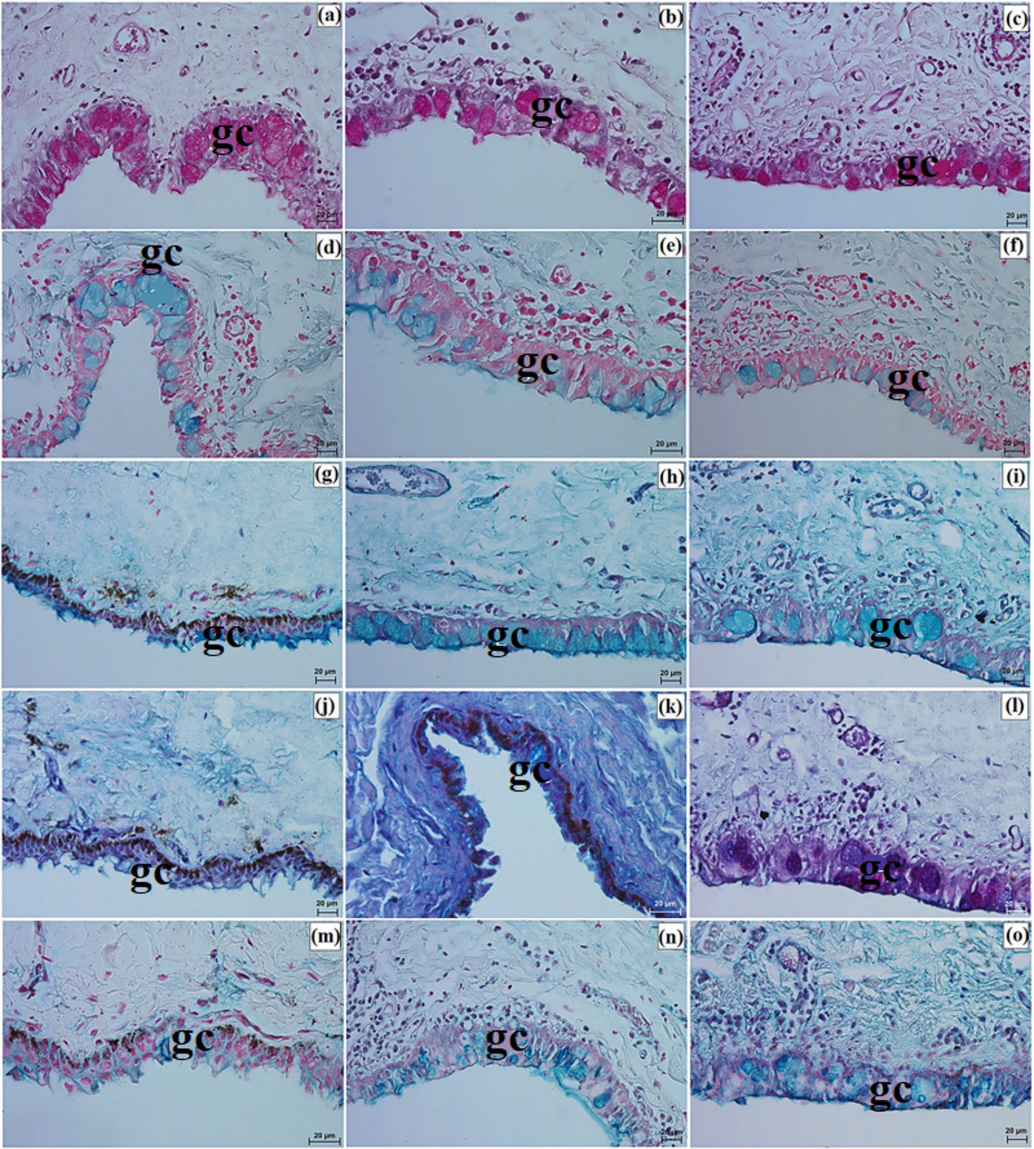
Micrographs of the histochemistry of goblet cells (gc) in the epithelium of the eyelids of the clouded leopard (*Neofelis nebulosa*) (**a**, **d**, **g**, **j**, **m**), the Angola lions (*Panthera leo bleyenberghi*) (**b**, **e**, **h**, **k**, **n**), and the Pallas’s cat (*Otocolobus manul manul*) (**c**, **f**, **i**, **l**, **o**). a – c = PAS stain; d – f = AB pH 1.0 stain; g – i = AB pH 2.5 stain; j – l = AB pH 2.5/PAS stain; m – o = CI stain. Scale bars: a – o = 20 μm. Magnifications: 40x – a, b, c, d, e, f, g, h, i, j, k, l, m, n, o

Elements of conjunctiva-associated lymphoid tissue were observed in all examined taxa, but their distribution differed. In the Pallas’s cat, lymphoid follicles, diffuse lymphocytes, and high endothelial venules were present in both eyelids, whereas in the clouded leopard and Angola lions these elements were identified only in the lower eyelid (Fig. 8p and s). Histochemically, the tarsal and sebaceous glands were negative in all examined individuals, whereas weak proteinaceous staining was observed in the sweat glands (Fig. 9a and o; Table 7). In contrast, goblet cells showed strong staining with all applied methods in the clouded leopard, Angola lions, and Pallas’s cat (Fig. 10a and o; Table 7).

**Table 7:** Histochemical analysis of eyelid glands; goblet cells of the upper and lower eyelid conjunctiva; superficial gland of the third eyelid; goblet cells of the third eyelid conjunctiva; lacrimal gland; and goblet cells of the intralobar ducts of the lacrimal gland in clouded leopard (*n* = 1), Angola lions (male, *n* = 1 and female, *n* = 1), and Pallas’s cat (*n* = 1)

Staining method	Sweat glands			Sebaceous glands and tarsal glands			Goblet cells of the upper and lower eyelids conjunctiva			Superficial gland of the third eyelid			Goblet cells of the third eyelid conjunctiva			Lacrimal gland			Goblet cells of the intralobar ducts of the lacrimal gland		
	Clouded leopard female	Angola lions (male and female	Pallas’s cat male	Clouded leopard female	Angola lions (male and female)	Pallas’s cat male	Clouded leopard female	Angola lions (male and female)	Pallas’s cat male	Clouded leopard female	Angola lions (male and female)	Pallas’s cat male	Clouded leopard male	Angola lions (male and female)	Pallas’s cat male	Clouded leopard female	Angola lions (male and female)	Pallas’s cat male	Clouded leopard female	Angola lions (male and female)	Pallas’s cat male
PAS	–/+	–	–/+	–			+++	+++	+++	+++	–	++	+++	+++	+++	+++	+	+/++	+++	+++	+++
AB pH 1.0	+	–/+	+	–			+	+	–/+	–/+	–	+/++	+	+	+	++	+	–/+	+++	++	+
AB pH 2.5	+	+++	–/+	–			+++	+++	++	++	+	+++	++/+++	++/+++	+++	+++	–/+	++	+++	–/+	+
AB pH 2.5/ PAS	+ (magenta color)	+ (blue color)	–/+ (blue color)	–			+++ (blue color)	dominant +/++ (blue color) and sparse –/+ (magenta color)	dominant +++ (blue color) and sparse –/+ (magenta color)	dominant ++ (magenta color) and sparse ++ (blue color)	++ (blue color)	dominant + (blue color) and sparse +++ (magenta color)	dominant + (magenta color) and sparse ++ (blue color)	+++ (blue color)	dominant +++ (magenta color) and sparse ++ (blue color)	+++ (blue color)	++ (blue color)	++ (blue color) and + (magenta color)	dominant +++ (blue color) and sparse ++ (magenta color)	+++ (blue color)	+++ (magenta color)
CI	++	+++	+	–			+/++	++/+++	+/++	+++	+	++/+++	+/++	+++	+/++	++/+++	+	++	+++	+	++

### The superficial gland of the third eyelid morphology

The morphometric analysis of the superficial gland of the third eyelid (SGTE), with results presented in Table 6, showed differences in gland dimensions among the examined individuals. The largest SGTE was observed in the Angola lions, the smallest in the Pallas’s cat, and an intermediate size in the clouded leopard (Table 6). In all examined individuals, SGTE had a flattened shape. In the clouded leopard and Pallas’s cat, it appeared more rounded or broadly oval, whereas in the Angola lions it was more elongated. In the Angola lions, the male showed slightly larger SGTE dimensions than the female (Table 6). SGTE in all examined species was located in the medial canthus of the eye between the medial and ventral rectus muscles and was partially covered by the ventral oblique muscle (Fig. 6j and l).

**Fig. 11:**
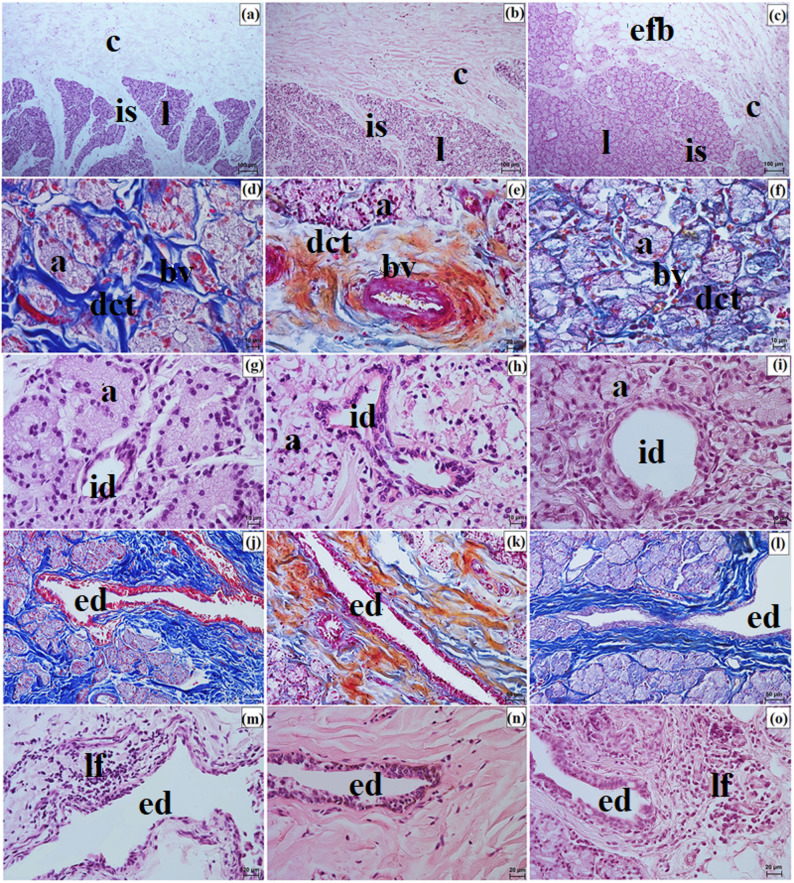
Micrographs of the superficial gland of the third eyelid (sgte) in the clouded leopard (*Neofelis nebulosa*) (**a**, **d**, **g**, **j**, **m**), the Angola lions (*Panthera leo bleyenberghi*) (**b**, **e**, **h**, **k**, **n**), and the Pallas’s cat (*Otocolobus manul manul*) (**c**, **f**, **i**, **l**, **o**). a – acini, bv – blood vessels, c – capsule, ed – excretory duct, efb – extraperiorbital fat body, id – intercalated duct, is – interlobar septa, l – lobes, lf – lymphoid follicle. a – c, g – i, m – o = Mayer’s H&E stain; d, f, j = Mallory trichrome with aniline blue stain; e, k = Heidenhain’s Azan trichrome stain. Scale bars: a – c = 100 μm; j – l = 50 μm; e, m – o = 20 μm; d, f – i = 10 μm. Magnifications: 10x – a, b, c; 20x – d, j, k, l; 40x – e, m, n, o; 63x – f, g, h, i

**Fig. 12:**
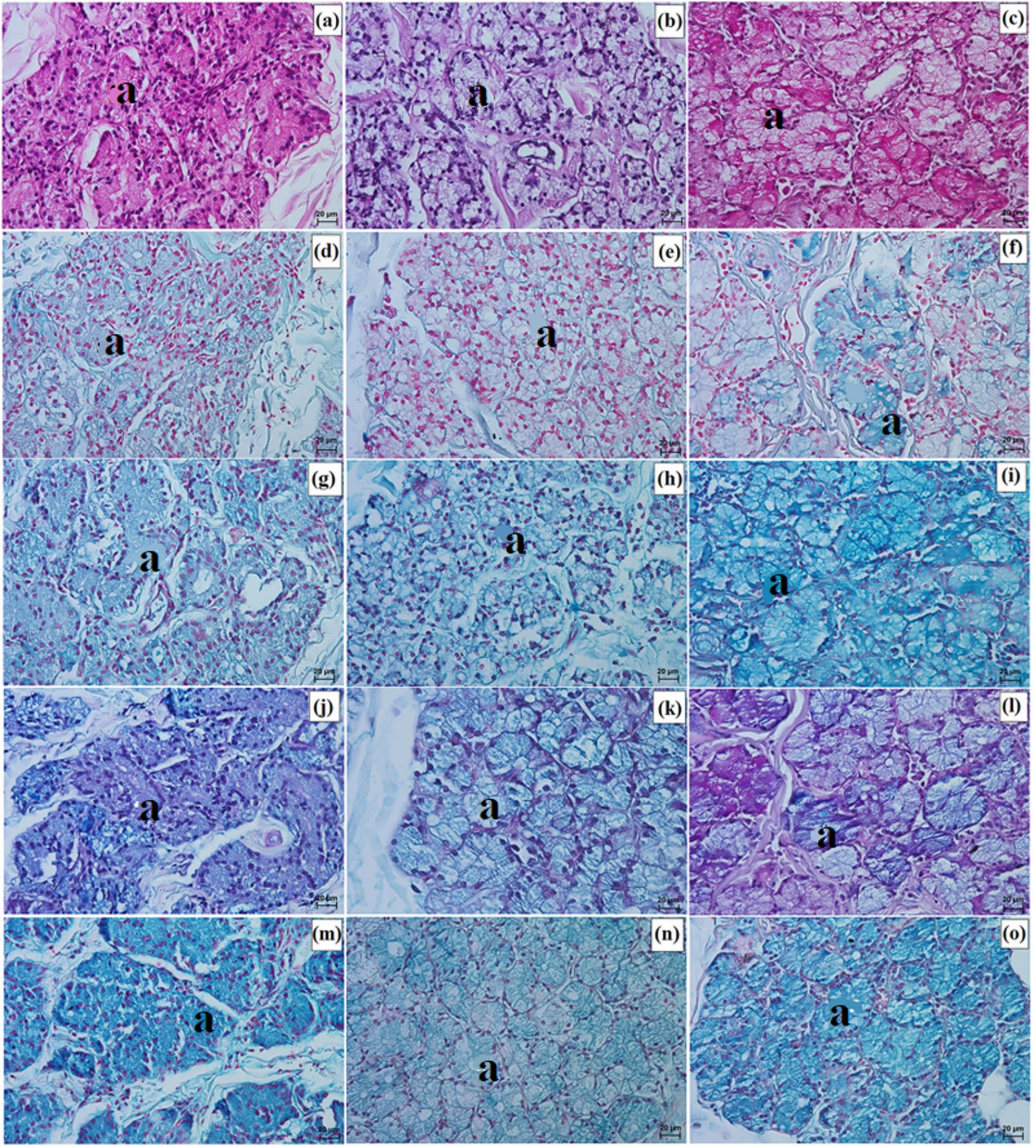
Micrographs of the histochemistry of superficial gland of the third eyelid (sgte) in the clouded leopard (*Neofelis nebulosa*) (**a**, **d**, **g**, **j**, **m**), the Angola lions (*Panthera leo bleyenberghi*) (**b**, **e**, **h**, **k**, **n**), and the Pallas’s cat (*Otocolobus manul manul*) (**c**, **f**, **i**, **l**, **o**). a – acini. a – c = PAS stain; d – f = AB pH 1.0 stain; g – i = AB pH 2.5 stain; j – l = AB pH 2.5/PAS stain; m – o = CI stain.Scale bars: a – o = 20 μm. Magnifications: 40x – a, b, c, d, e, f, g, h, i, j, k, l, m, n, o

SGTE was surrounded by an intraperiorbital fat body and enclosed by a connective tissue capsule, which was thicker in the clouded leopard and Angola lions and thinner in the Pallas’s cat. Interlobar septa extending from the capsule were thick in the Angola lions, thin in the Pallas’s cat, and variable in the clouded leopard. SGTE consisted predominantly of small lobes in the clouded leopard, whereas in the Angola lions and the Pallas’s cat, medium and large lobes predominated (Fig. 11a and c). The connective tissue capsule and interlobar septa were composed mainly of elastic fibers interwoven with collagen and reticular fibers and contained numerous blood vessels and nerves (Fig. 11d and f). In all examined individuals, SGTE showed a multilobar compound acinar structure with serous acini composed of conical cells and containing a small lumen. The intercalated ducts, located within the glandular lobes, were lined by a single layer of cuboidal epithelium (Fig. 11g and i). Within the interlobar septa, numerous excretory ducts lined by a single layer of columnar epithelium were present. Clusters of lymphocytes around these ducts were observed in the clouded leopard and Pallas’s cat (Fig. 11j and o). Histochemically, SGTE in the examined felids exhibited a serous character (Fig. 12a and o; Table 7).

### The third eyelid morphology

The measurement results of the length of the upper and lower branches of the third eyelid (TE), and the length of the crossbar of TE, are presented in Table 6. Only small differences were observed among the examined individuals, including between the male and female Angola lions and between the clouded leopard and the Pallas’s cat (Table 6). TE of the examined Felidae was T-shaped. The palpebral conjunctiva covering the free margin of TE was highly pigmented in these species (Fig. 6m and o).

**Fig. 13:**
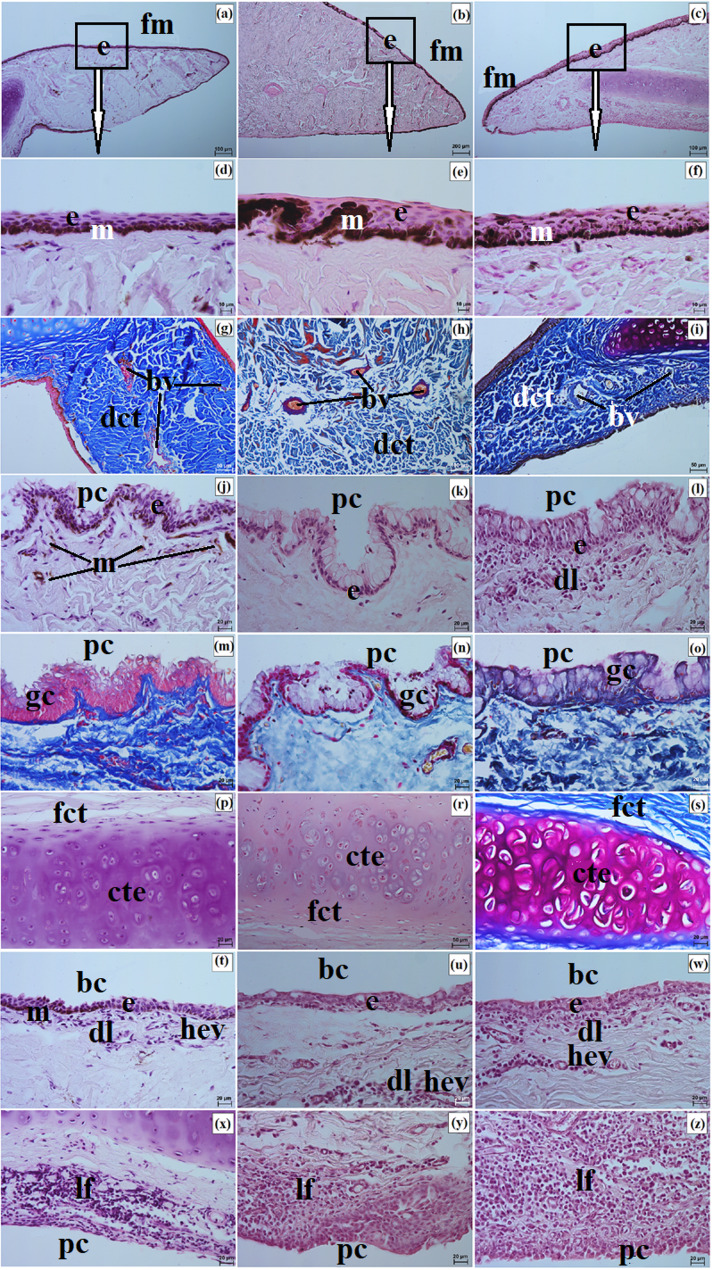
Micrographs of the third eyelid (te) in the clouded leopard (*Neofelis nebulosa*) (**a**, **d**, **g**, **j**, **m**, **p**, **t**, **x**), the Angola lions (*Panthera leo bleyenberghi*) (**b**, **e**, **h**, **k**, **n**, **r**, **u**, **y**), and the Pallas’s cat (*Otocolobus manul manul*) (**c**, **f**, **i**, **l**, **o**, **s**, **w**, **z**). bc – bulbar conjunctiva, bv – blood vessels, cte – cartilage of the third eyelid, dct – dense connective tissue, dl – diffuse lymphocytes, e – epithelium, fct – fibrous connective tissue, fm – free margin, gc – goblet cells, hev – high endothelial venules, lf – lymphoid follicle, m – melanocytes, pc – palpebral conjunctiva. a – f, j – l, p, r, t – z = Mayer’s H&E stain; h, i, n, o, s = Mallory trichrome with aniline blue stain; g, m = Heidenhain’s Azan trichrome stain. Scale bars: b = 200 μm; a, c, h = 100 μm; g, i, r = 50 μm; j – p, s – z = 20 μm; d – f = 10 μm. Magnifications: 5x – b; 10x – a, c, h; 20x – d, g, i, r; 40x – j, k, l, m, n, o, p, s, t, u, w, x, y, z; 63x – e, f

The palpebral conjunctiva of TE was covered with nonkeratinized stratified columnar epithelium, consisting of 6 to 8 layers in the clouded leopard, 8 to 10 in the Angola lions, and 4 to 5 in the Pallas’s cat (Fig. 13a and f). The free margin of TE was composed of dense fibrous connective tissue with an irregular arrangement and numerous blood vessels (Fig. 13g and i). In the clouded leopard, numerous melanin granules were visible beneath the palpebral conjunctival epithelium (Fig. 13j and l). This epithelium contained numerous goblet cells (Fig. 13m and o). The cartilage of TE was surrounded by a thick layer of collagen, reticular, and elastic fibers and was composed of elastic cartilage in all the examined Felidae (Fig. 13p and s). The bulbar conjunctiva of TE was lined by non-keratinized stratified cuboidal epithelium, with 5 to 7 layers in the clouded leopard and Angola lions and 3 to 4 layers in the Pallas’s cat; goblet cells were present in all examined individuals (Fig. 13t and w). Both the palpebral and bulbar conjunctiva contained conjunctival lymphoid nodules, diffuse lymphocytes, and high endothelial venules (Fig. 13t and z).

**Fig. 14:**
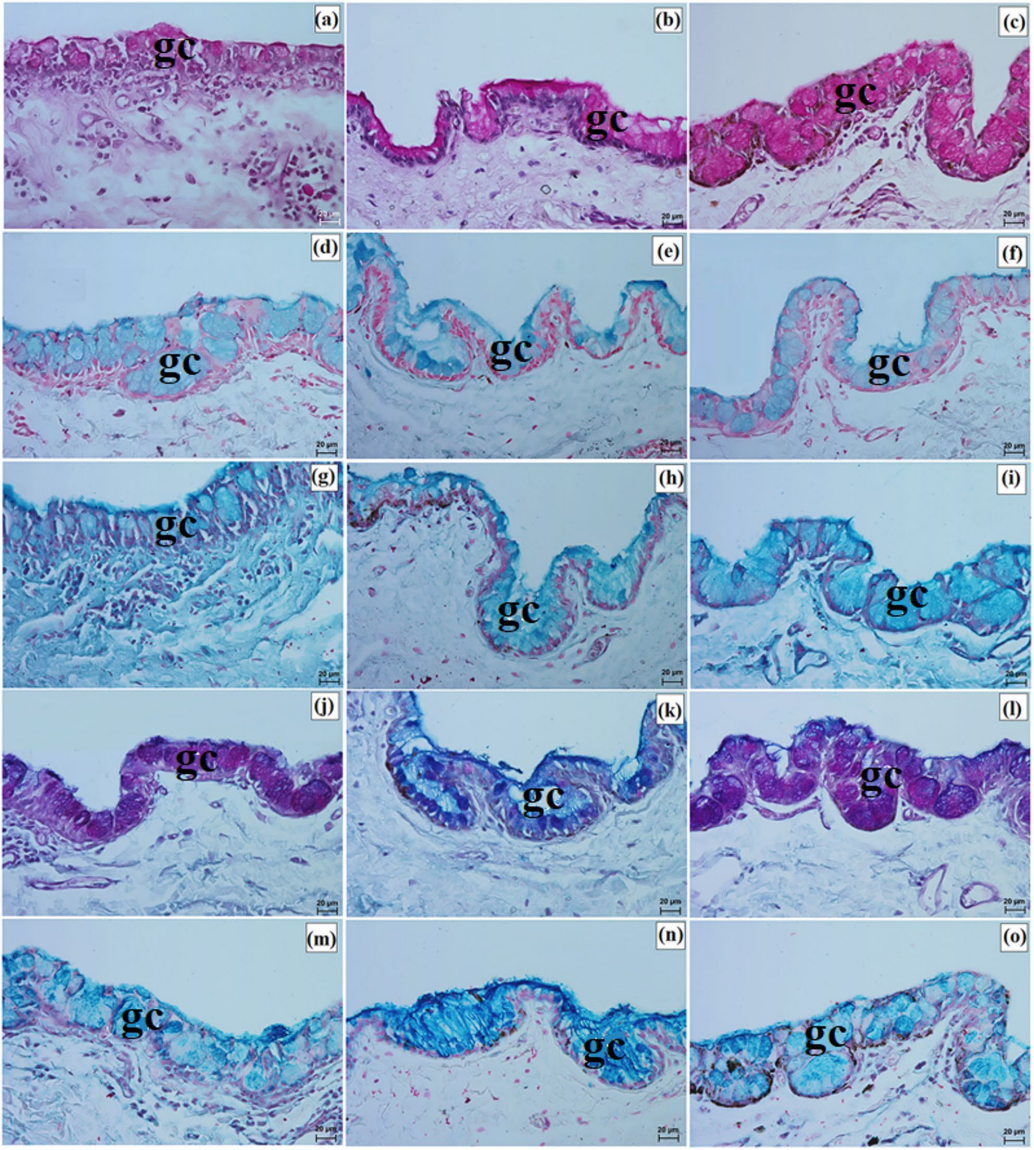
Micrographs of the histochemistry of goblet cells (gc) in the epithelium of the third eyelid (te) of the clouded leopard (*Neofelis nebulosa*) (**a**, **d**, **g**, **j**, **m**), the Angola lions (*Panthera leo bleyenberghi*) (**b,** **e**, **h**, **k**, **n**), and the Pallas’s cat (*Otocolobus manul manul*) (**c**, **f**, **i**, **l**, **o**). a – c = PAS stain; d – f = AB pH 1.0 stain; g – i = AB pH 2.5 stain; j – l = AB pH 2.5/PAS stain; m – o = CI stain. Scale bars: a – o = 20 μm. Magnifications: 20x – a, b, c, d, e, f, g, h, i, j, k, l, m, n, o

The histochemical results showed that goblet cells were strongly stained using PAS, AB pH 1.0, AB pH 2.5, and CI. Staining with AB pH 2.5/PAS revealed that in the clouded leopard and Pallas’s cat, goblet cells predominantly appeared magenta, whereas in the Angola lions they were predominantly blue (Fig. 14a and o; Table 7).

### The lacrimal gland morphology

The measurement results of the lacrimal gland (LG) in all the examined Felidae are presented in Table 6. Differences in LG length, width, and thickness were observed among the examined individuals (Table 6). In the examined Felidae, LG was oval or ellipsoidal, slightly flattened in shape, and light pink in color (Fig. 6p and s).

**Fig. 15:**
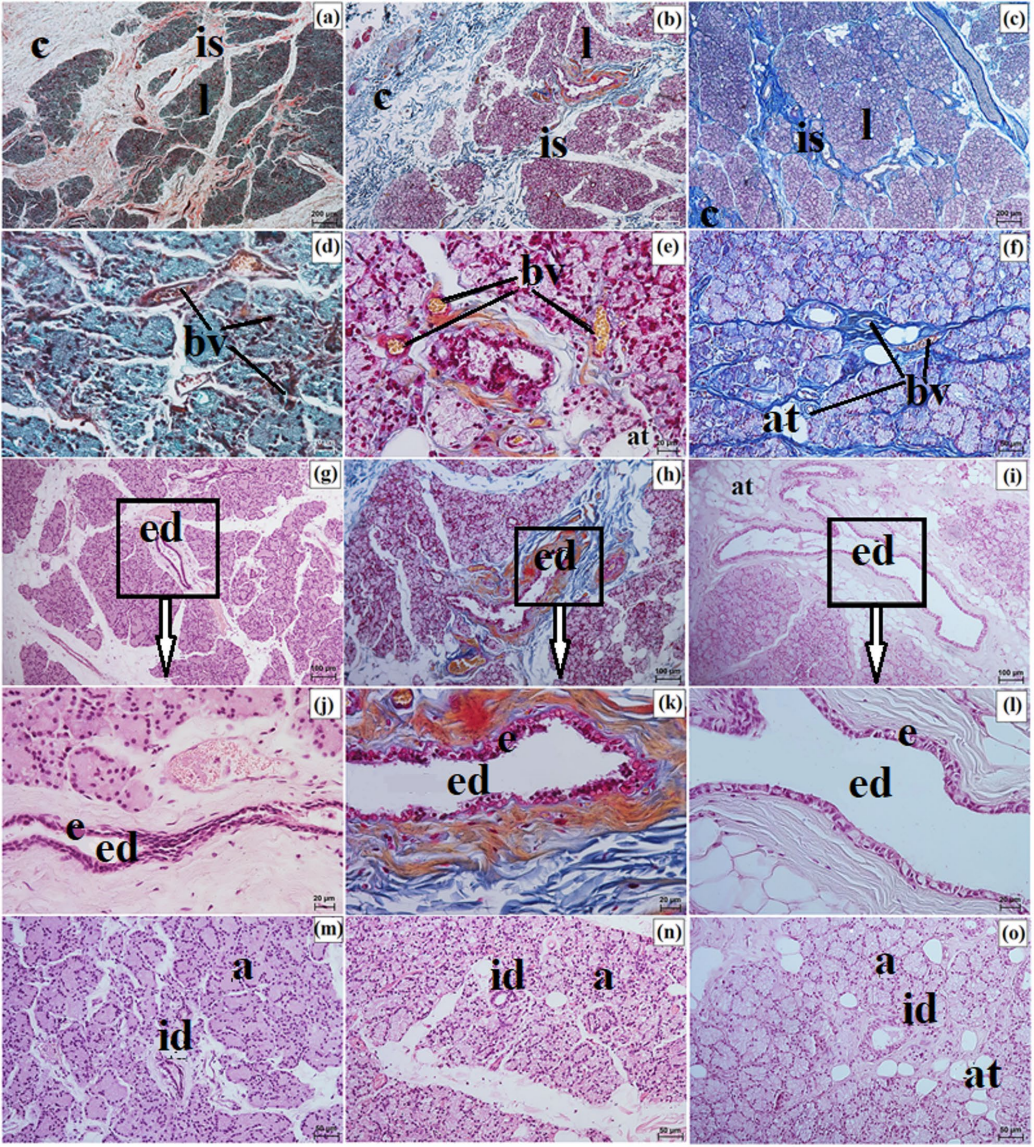
Micrographs of the lacrimal gland (lg) in the clouded leopard (*Neofelis nebulosa*) (**a, d, g, j, m**), the Angola lions (*Panthera leo bleyenberghi*) (**b, e, h, k, n**), and the Pallas’s cat (*Otocolobus manul manul*) (**c, f, i, l, o**). a – acini, at – adipose tissue, bv – blood vessels, c – capsule, e – epithelium, ed – excretory duct, id – intercalated duct, is – interlobar septa, l – lobes. a, d = Movat pentachrome (modified Russell Movat) stain; b, e, h, k = Mallory trichrome with aniline blue stain; c, d = Heidenhain’s Aazan trichrome stain; g, i, j, l – o = Mayer’s H&E stain. Scale bars: a – c = 200 μm; g – i = 100 μm; f, m – o = 50 μm; d, e, j – l = 20 μm. Magnifications: 5x – a, b, c; 10x – g, h, i; 20x – f, m, n, o; 40x – d, e, j, k, l

**Fig. 16:**
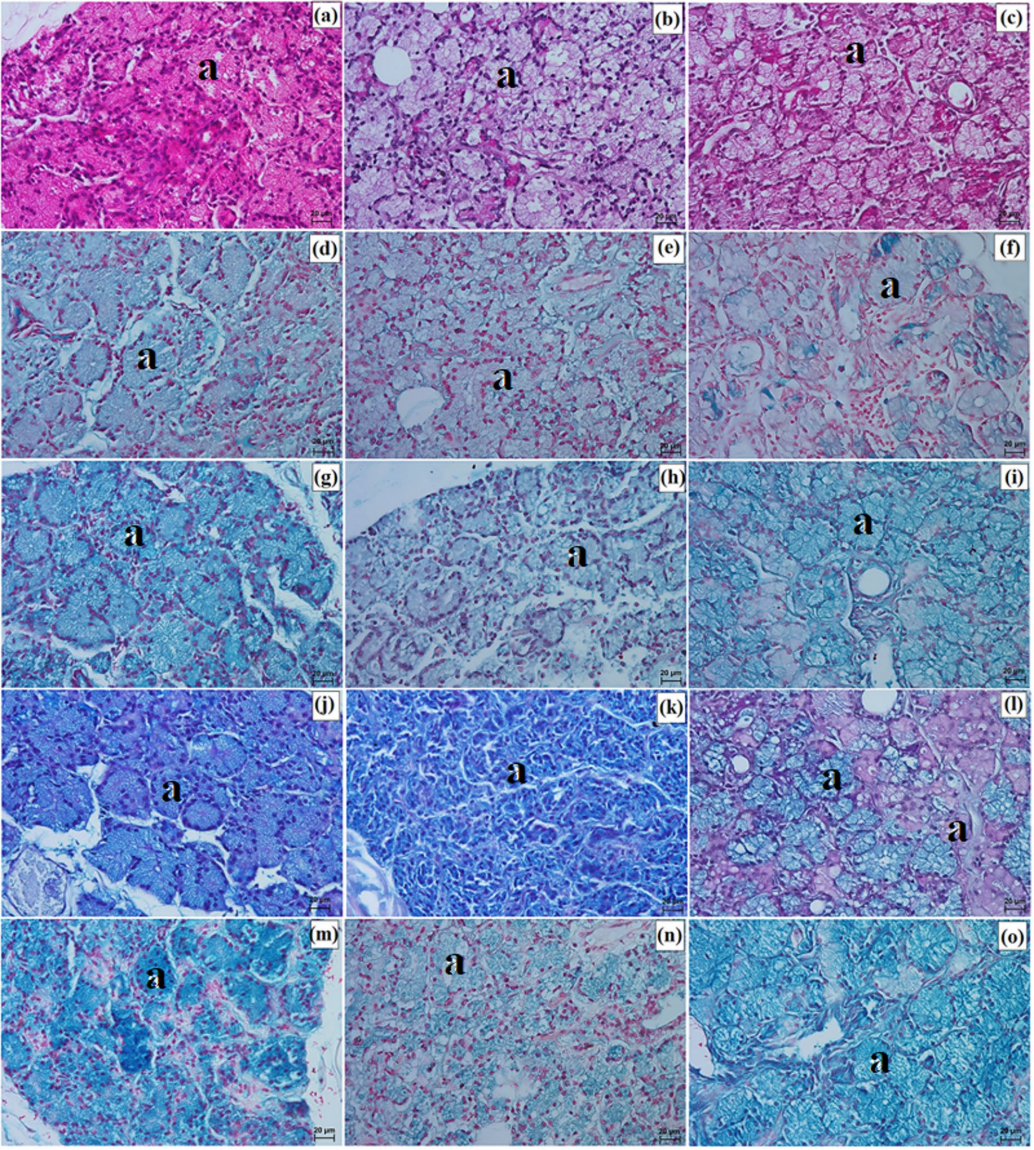
Micrographs of the histochemistry of lacrimal gland (lg) in the clouded leopard (*Neofelis nebulosa*) (**a, d, g, j, m**), the Angola lions (*Panthera leo bleyenberghi*) (**b, e, h, k, n**), and the Pallas’s cat (*Otocolobus manul manul*) (**c, f, i, l, o**). a – acini. a – c = PAS stain; d – f = AB pH 1.0 stain; g – i = AB pH 2.5 stain; j – l = AB pH 2.5/PAS stain; m – o = CI stain. Scale bars: a – o = 20 μm. Magnifications: 40x – a, b, c, d, e, f, g, h, i, j, k, l, m, n, o

**Fig. 17:**
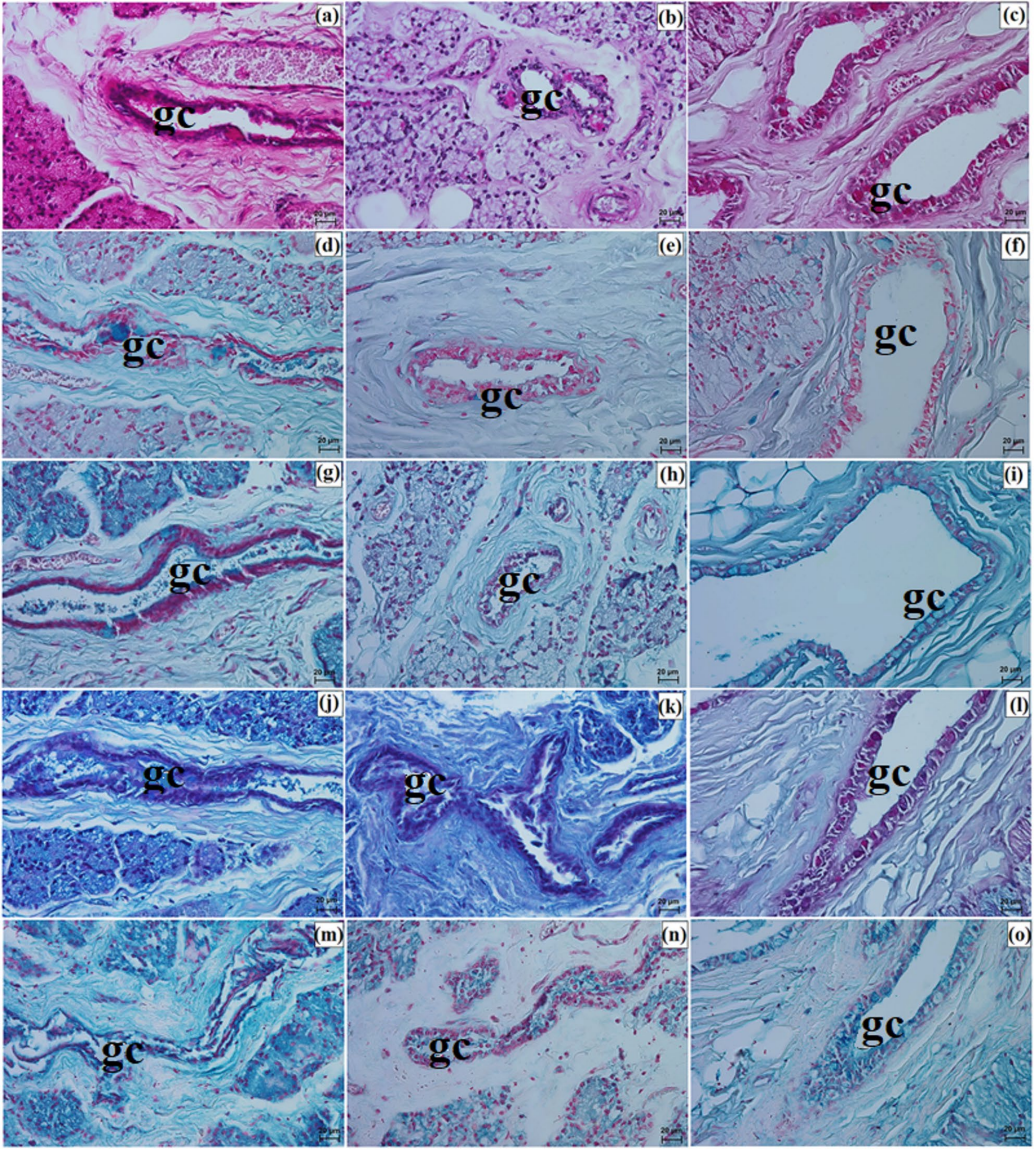
Micrographs of the goblet cells (gc) in the excretory duct of the lacrimal gland (lg) in the clouded leopard (*Neofelis nebulosa*) (**a, d, g, j, m**), the Angola lions (*Panthera leo bleyenberghi*) (**b, e, h, k, n**), and the Pallas’s cat (*Otocolobus manul manul*) (**c, f, i, l, o**). a – c = PAS stain; d – f = AB pH 1.0 stain; g – i = AB pH 2.5 stain; j – l = AB pH 2.5/PAS stain; m – o = CI stain. Scale bars: a – o = 20 μm. Magnifications: 40x – a, b, c, d, e, f, g, h, i, j, k, l, m, n, o

LG in the clouded leopard and Angola lions (male and female) was surrounded by a thick connective tissue capsule (dense irregular connective tissue) (Fig. 15a and b), whereas in the Pallas’s cat, the capsule was thin (Fig. 15c). The capsule formed thick septa, along with numerous large and small lobes in the clouded leopard and Angola lions (male and female) (Fig. 15a and b), while in the Pallas’s cat, thin interlobar septa divided the gland into several large lobes and a few medium and small lobes (Fig. 15c). In all the animals, the connective tissue contained numerous blood vessels with both small and large diameters (Fig. 15d and f). Numerous excretory ducts were observed in the interlobar septa, lined with a single layer of columnar epithelium, containing many goblet cells (Fig. 15g and l). LG had a multilobar acinar structure, with acini containing small lumens and composed of tall conical secretory cells (Fig. 15m and o). In the Pallas’s cat, adipose cells were observed in the interlobular septa and acini (Fig. 15o). Numerous intercalated ducts in all the examined Felidae were lined with a single layer of cuboidal epithelium (Fig. 15m and o). Histochemical analysis revealed that moderate staining predominated in the secretory units and goblet cells of the intralobar ducts across all methods. In all three examined species, the LG exhibited a serous character (Figs. 16a and o and 17a and o; Table 7).

### The orbital region anatomy

The following orbital parameters are presented in Tables 8 and 9. The orbital index varied among the examined skulls and also between the left and right sides in some individuals. In the female clouded leopard, the left orbit (LO) had a higher orbital index than the right orbit (RO). In the two male Pallas’s cats, the specimen identified as *Otocolobus manul manul* had a higher orbital index than *Otocolobus manul*, and left-right asymmetry was also present in both individuals. In the female and male Angola lions, the orbital index was below 100%, indicating that the orbits were wider than high. Additional variation in orbital index and left-right asymmetry was also observed in the African lion skulls (Table 9).

**Table 8:** Morphometry (mm) (mean ± standard deviation) of orbital parameters in clouded leopard (female, *n* = 1), and Pallas’s cat (male, *n* = 2)

Parameters	Side	Clouded leopard (*Neofelis nebulosa*)		Pallas’s cat (*Otocolobus manul manul*)		Pallas’s cat (*Otocolobus manul*)	
		Mean	SD	Mean	SD	Mean	SD
Orbital vertical length	**LO**	31.816	± 0.05	24.76	± 0.03	25.9	± 0.1
	**RO**	33.073	± 0.1	24.6	± 0.05	25.73	± 0.2
Orbital horizontal width	**LO**	31.543	± 0.06	27.88	± 0.06	27.92	± 0.5
	**RO**	31.56	± 0.3	26.94	± 0.03	27.54	± 0.03
Orbital index (%)	**LO**	99.16	± 0.2	112.6	± 0.2	107.78	± 1.9
	**RO**	95.43	± 1.1	109.46	± 0.2	106.85	± 0.9
Orbital depth	**LO**	46.203	± 0.05	35.73	± 0.3	36.15	± 0.6
	**RO**	46.813	± 0.1	35.6	± 0.7	36.38	± 0.64
Orbital area (mm^2^)	**LO**	788.5	± 2.5	548.28	± 1.2	567.19	± 12.2
	**RO**	819.65	± 9.1	520.21	± 1.3	556.43	± 4.22
Interorbital distance:	**At the rostral level**	30.626	± 0.3	23.39	± 0.05	25.97	± 0.09
	**At the middle level**	30.551	± 0.2	31.01	± 0.06	34.18	± 0.3
	**At the caudal level**	73.086	± 0.07	63.53	± 0.2	68.42	± 0.3
Frontal length	**LO**	26.583	± 0.06	28.68	± 0.3	28.99	± 0.08
	**RO**	26.308	± 0.05	28.98	± 0.1	29.31	± 0.1
Lacrimal length	**LO**	12.866	± 0.1	8.68	± 0.1	7.8	± 0.2
	**RO**	13.136	± 0.04	7.85	± 0.2	6.88	± 0.1
Malar length	**LO**	29.48	± 0.09	27.57	± 0.1	26.00	± 0.4
	**RO**	30.418	± 0.5	27.29	± 0.2	27.26	± 0.1

*LO* Left orbit, *RO* Right orbit

**Table 9:** Morphometry (mm) (mean ± SD) of orbital parameters in African lions (male, *n* = 1; no sex information, *n* = 6) and Angola lions (male, *n* = 3 and female, *n* = 1)

Parameters	Side	African lion (*Panthera leo*)									Angola lion (*Panthera leo bleyenberghi*)	
		BPI/C/183 NSI	BPI/C/184 NSI	BPI/C/185 NSI	BPI/C/186 male	BPI/C/187 NSI	ZA17 NSI	BPI/4/1351 NSI	FE/PL/1 male	FE/PL/2 male	FE/PLB/1 female	FE/PLB/2 male
Orbital vertical length	**LO**	61.09 ± 0.7	57.77 ± 0.9	56.39 ± 0.4	60.54 ± 1.5	59.11 ± 0.8	73.70 ± 0.4	73.86 ± 0.6	70.99 ± 0.8	65.70 ± 0.9	58.00 ± 0.1	62.00 ± 0.09
	**RO**	62.86 ± 0.6	57.91 ± 0.3	56.87 ± 0.1	62.75 ± 0.2	60.03 ± 0.9	74.66 ± 0.6	74.94 ± 0.7	68.478 ± 0.5	65.17 ± 0.9	58.55 ± 0.2	62.2 ± 0.05
Orbital horizontal width	**LO**	41.165 ± 1.4	41.34 ± 0.5	43.07 ± 1.1	45.01 ± 0.5	40.91 ± 0.5	44.53 ± 1.2	48.15 ± 1.1	54.96 ± 0.7	52.39 ± 0.7	54.913 ± 0.4	55.41 ± 0.3
	**RO**	41.95 ± 0.2	41.11 ± 0.8	41.07 ± 0.8	40.82 ± 1.1	41.03 ± 1.1	45.9 ± 0.5	49.64 ± 1.1	55.31 ± 0.4	52.70 ± 0.5	51.80 ± 0.2	56.43 ± 0.4
Orbital index (%)	**LO**	67.396 ± 2.4	71.57 ± 0.9	76.4 ± 2.5	74.42 ± 2.54	69.22 ± 1.6	60.41 ± 1.5	65.21 ± 1.4 2	77.44 ± 0.6	79.76 ± 0.9	94.66 ± 0.8	89.38 ± 0.6
	**RO**	66.74 ± 1.2	70.99 ± 1.7	72.22 ± 1.6	65.05 ± 1.9	68.37 ± 2.4	61.81 ± 1.1	66.25 ± 1.8	80.78 ± 0.7	80.88 ± 1.4	88.46 ± 0.3	90.73 ± 0.7
Orbital depth	**LO**	76.63 ± 0.5	79.7 ± 0.9	79.08 ± 1.5	80.58 ± 1.5	72.45 ± 3.2	93.82 ± 0.8	103.44 ± 1.1	88.72 ± 1.6	85.17 ± 0.4	80.67 ± 0.4	77.03 ± 0.4
	**RO**	78.011 ± 1.0	80.48 ± 0.5	81.06 ± 1.1	81.51 ± 1.2	74.30 ± 1.9	96.08 ± 1.4	106.66 ± 1.1	87.27 ± 0.6	86.22 ± 0.4	79.12 ± 0.5	78.74 ± 0.4
Orbital area (mm^2^)	**LO**	1975.01 ± 71.6	1876.48 ± 51.1	1908.08 ± 41.9	2138.58 ± 59.2	1900.16 ± 43.3	2575.3 ± 89.0	2794.12 ± 77.3	3064.34 ± 72.9	2704.8 ± 71.8	2500.24 ± 19.1	2700.01 ± 23.5
	**RO**	2071.36 ± 25.4	1869.92 ± 35.7	1835.24 ± 38.1	2011.23 ± 54.6	1935.54 ± 43.1	2689.93 ± 38.6	2922.28 ± 59.9	2977.14 ± 41.7	2698.4 ± 52.1	2366.97 ± 29.6	2757.6 ± 22.5
Interorbital distance:	**At the rostral level**	67.42 ± 0.3	67.59 ± 0.3	n.d.	69.58 ± 0.3	62.12 ± 0.6	85.65 ± 0.2	97.49 ± 0.6	83.65 ± 0.6	84.27 ± 0.6	79.05 ± 0.1	91.46 ± 0.4
	**At the middle level**	90.65 ± 0.2	91.15 ± 0.04	96.34 ± 0.1	n.d.	82.23 ± 0.09	115.56 ± 0.2	130.63 ± 0.07	83.00 ± 0.5	100.49 ± 1.1	96.05 ± 0.3	97.56 ± 0.2
	**At the caudal level**	144.67 ± 0.5	141.68 ± 0.7	145.95 ± 2.4	155 0 0	140.8 ± 0.8	175 0 0	185 0 0	162.88 ± 1.2	159.17 ± 0.5	154.86 ± 0.4	179.46 ± 0.2
Frontal length	**LO**	53.445 ± 0.7	55.16 ± 0.7	n.d.	55.16 ± 0.9	54.64 ± 0.8	68.74 ± 0.6	n.d.	57.58 ± 0.9	55.83 ± 0.3	50.56 ± 0.3	48.36 ± 0.3
	**RO**	55.416 ± 0.8	53.01 ± 1.1	n.d.	n.d.	52.513 ± 1,8	66.03 ± 0.9	n.d.	60.58 ± 0.4	61.34 ± 0.6	49.84 ± 0.1	48.13 ± 0.3
Lacrimal length	**LO**	27.02 ± 0.3	2.66 ± 0.6	n.d.	22.04 ± 0.8	24.39 ± 1.1	29.87 ± 0.3	n.d.	28.11 ± 0.5	28.99 ± 0.6	26.51 ± 0.3	28.87 ± 0.1
	**RO**	27.11 ± 0.4	21.78 ± 0.7	n.d.	n.d.	24.55 ± 0.3	28.7 ± 0.8	n.d.	25.81 ± 0.4	33.21 ± 0.4	28.60 ± 0.3	30.66 ± 0.4
Malar length	**LO**	52.47 ± 0.5	51.99 ± 0.5	n.d.	48.83 ± 0.5	43.29 ± 0.6	57.45 ± 0.5	n.d.	63.92 ± 0.8	61.795 ± 0.3	56.12 ± 0.4	56.44 ± 0.4
	**RO**	50.79 ± 0.3	54.27 ± 0.5	n.d.	n.d.	44.29 ± 0.4	58.53 ± 0.4	n.d.	61.04 ± 0.6	62.67 ± 0.6	54.95 ± 0.2	58.13 ± 0.2

*LO* Left orbit, *RO* Right orbit, *NSI* No sex information; n.d. – measurement not determined due to cranial damage

**Fig. 18:**
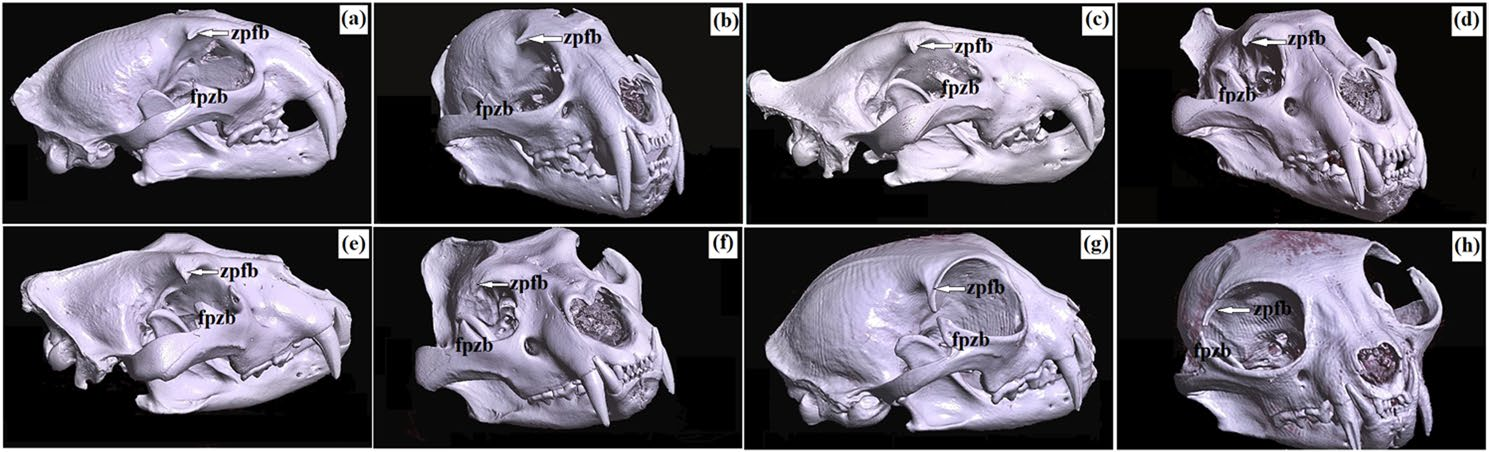
Three-dimensional reconstructed CT images of the head in the clouded leopard (*Neofelis nebulosa*) (**a**, **b**), the African lions (*Panthera leo*) (**c**, **d**), the Angola lions (*Panthera leo bleyenberghi*) (**e**, **f**), and the Pallas’s cat (*Otocolobus manul manul*) (**g**, **h**). Right lateral aspect (**a**, **c**, **e**, **g**) and right-rostro-lateral aspect (**b**, **d**, **f**, **h**). In the clouded leopard, African lions, and Angola lions, the frontal process of the zygomatic bone was prominent, with a well defined zygomatic process of the frontal bone. In Pallas’s cat, the zygomatic process of the frontal bone was much longer. fpzb – frontal process of zygomatic bone, zpfb – zygomatic process of frontal bone (white arrow)

**Fig. 19:**
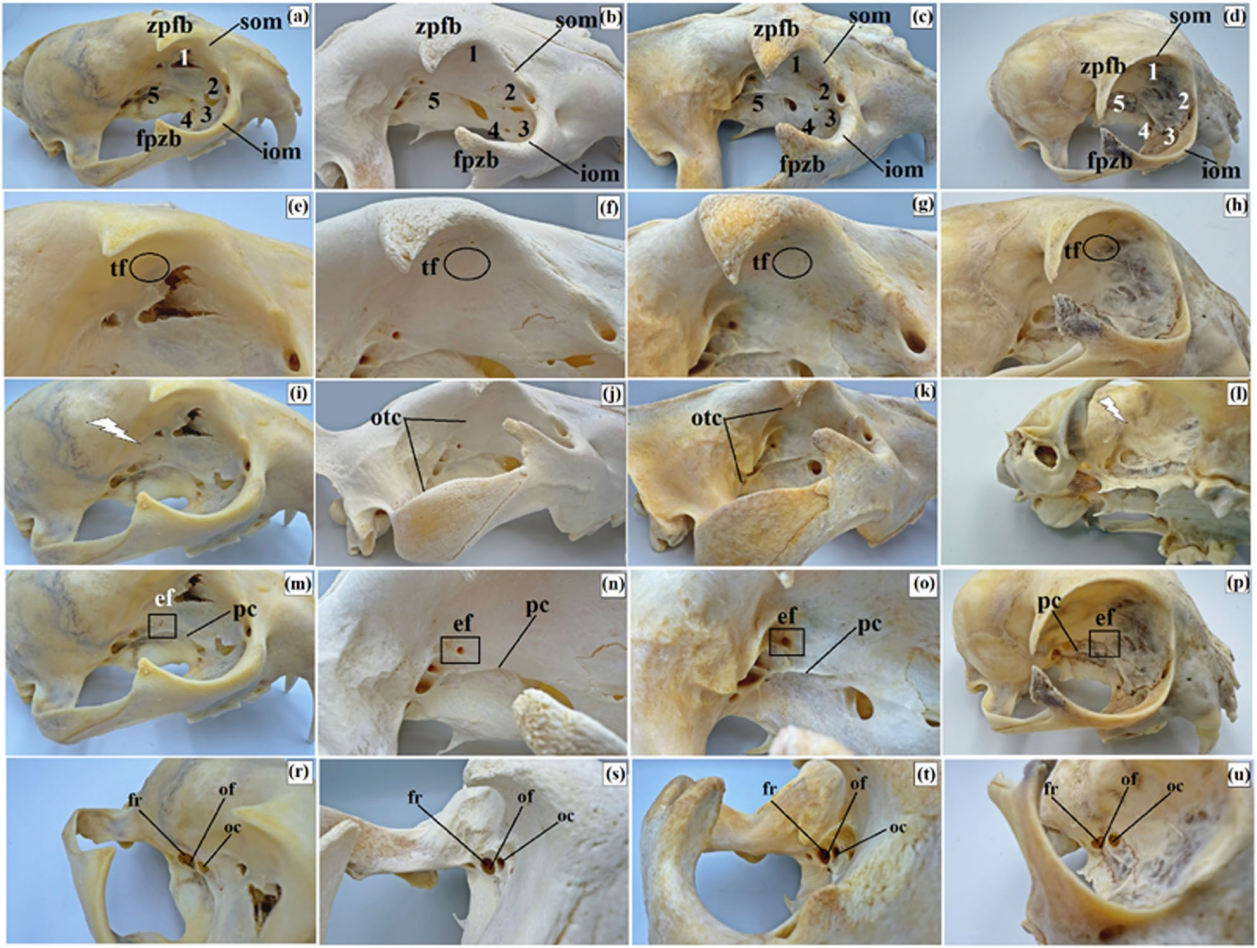
Macrographs of the orbit in the clouded leopard (*Neofelis nebulosa*) (**a, e, i, m, r**), the African lions (*Panthera leo*) (**b, f, j, n, s**), the Angola lions (*Panthera leo bleyenberghi*) (**c, g, k, o, t**), and the Pallas’s cat (*Otocolobus manul manul*) (**d, h, l, p, u**). 1 – orbital part of the frontal bone, 2 – orbital surface of the lacrimal bone, 3 – orbital surface of the body of maxilla, 4 – sphenoidal process of the palatine bone, 5 – pterygoid process of basisphenoid bone, black lightning bolt – absence of the orbitotemporal crest, ef – ethmoid foramen, fpzb – frontal process of the zygomatic bone, fr – foramen rotundum, iom – infraorbital margin, oc – optic canal, of – orbital fissure, otc – orbitotemporal crest, pc – pterygoid crest, som – supraorbital margin, tf – trochlea fovea, zpfb – zygomatic process of the frontal bone

The periorbita in the clouded leopard, African lions, Angola lions, and Pallas’s cats was conical in shape, and the orbit was of the open type (Figs. 18a and h and 19a and d). The orbital ring was completed by a long orbital ligament in the clouded leopard (22.666 mm ± 0.1 mm), African lions (36.98 mm ± 1.4 mm), and Angola lions (26.36 mm ± 1.7 mm), whereas in the Pallas’s cats this ligament was very short (2.83 mm ± 0.6 mm). This ligament connected the frontal process of the zygomatic bone with the zygomatic process of the frontal bone in the clouded leopard, African lions, and Angola lions. In Pallas’s cats, the zygomatic process of the frontal bone was relatively longer (Figs. 18a and h and 19a and d). The supraorbital and infraorbital margins were rounded in the clouded leopard, African lions, and Angola lions, but sharp in the Pallas’s cats. The bones forming the orbital region in the examined felids included the frontal, lacrimal, palatine, maxillary, and basisphenoid bones (Fig. 19a and d).

The trochlear fovea for the dorsal oblique muscle was very weakly marked in the examined material (Fig. 19e and h). In the African and Angola lions, the temporal surface of the frontal bone showed a sharp and well-defined orbitotemporal crest, whereas in the clouded leopard and Pallas’s cats this crest was not visible (Fig. 19i and l). The ethmoid foramina also differed among taxa: in the African and Angola lions, a single large ethmoid foramen was present below the crest, whereas in the clouded leopard and Pallas’s cats, two small ethmoid foramina were observed on the sphenofrontal suture (Fig. 19m and p). A large orbital fissure was visible directly below the optic canal in all examined taxa, although the pterygoid crest was more distinct in the African and Angola lions (Fig. 19m and u). Directly below the orbital fissure, a large foramen rotundum was present on the wings of the basisphenoid bone in all examined taxa (Fig. 19r and u).

**Fig. 20:**
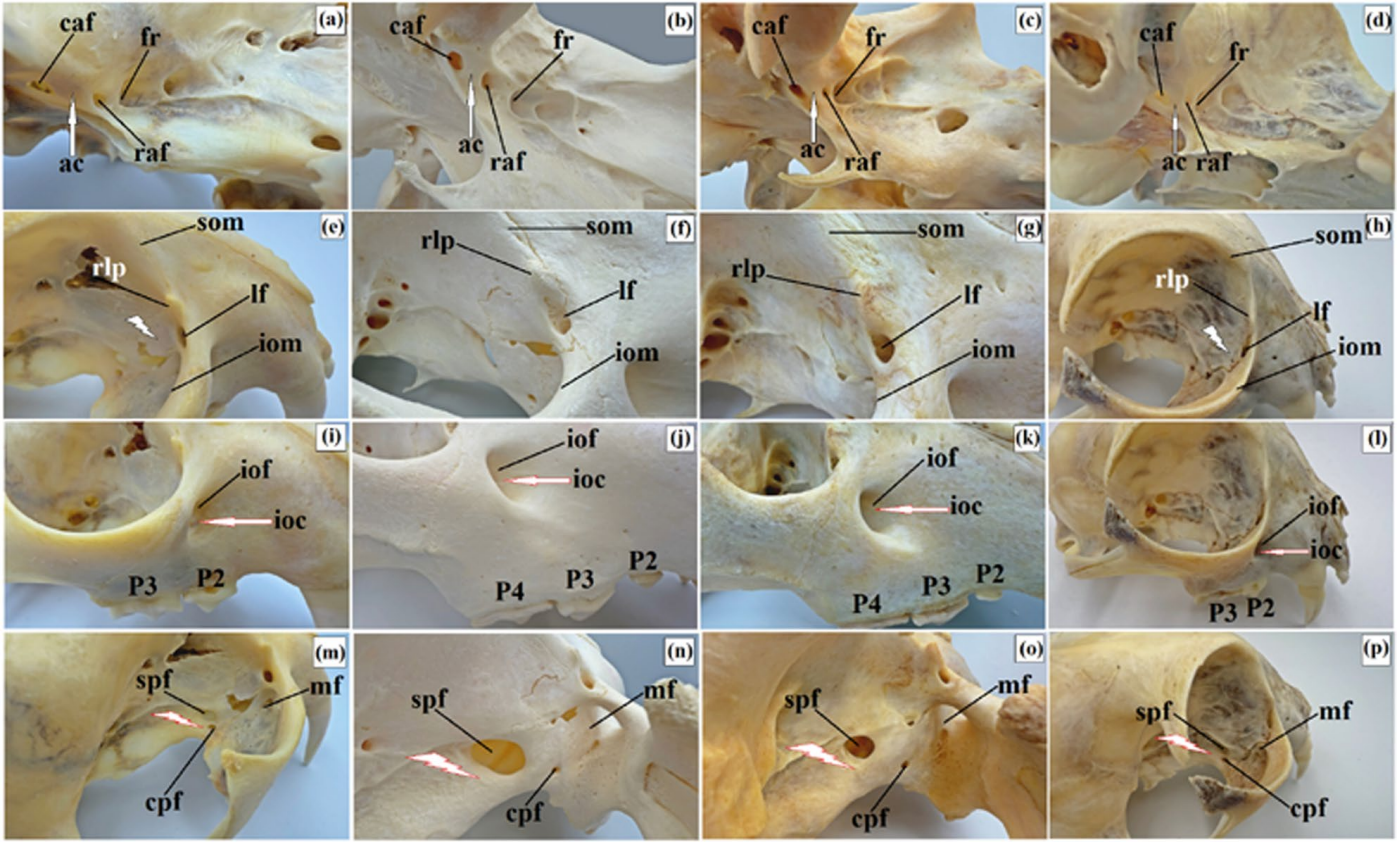
Macrographs of the orbit in the clouded leopard (*Neofelis nebulosa*) (**a, e, i, m**), the African lions (*Panthera leo*) (**b, f, j, n**), the Angola lions (*Panthera leo bleyenberghi*) (**c, g, k, o**), and the Pallas’s cat (*Otocolobus manul manul*) (**d, h, l, p**). ac – alar (pterygoid) canal (black arrows), black lightning bolt – orbital surface of the lacrimal bone, caf – caudal alar foramen, cpf – caudal palatine foramen, fr – foramen rotundum, ioc – infraorbital canal, iof – infraorbital foramen, iom – infraorbital foramen, lf – lacrimal foramen, mf – maxillary foramen, P2 – second premolar tooth, P3 – third premolar tooth, P4 – fourth premolar tooth, raf – rostral alar foramen, red lightning bolt – perpendicular plate of the palatine bone, rlp – rostral, lacrimal process, som – supraorbital margin, spf – sphenopalatine foramen

Further ventrally, the rostral alar foramen led through the alar (pterygoid) canal to the caudal alar foramen (Fig. 20a and d). The length of the alar canal was 6.85 mm (± 0.6 mm) in the clouded leopard, 6.85 mm (± 0.9 mm) in the African lions, 8.17 mm (± 0.5 mm) in the Angola lions, and 2.51 mm (± 0.5 mm) in the Pallas’s cats. The supraorbital foramen and the fossa of the ventral oblique muscle were absent in all examined taxa. On the rostral border between the supraorbital and infraorbital margins, a distinct rostral lacrimal process was present in the clouded leopard and Angola lions, whereas in African lions and Pallas’s cats it was less pronounced. The fossa for the lacrimal sac was weakly marked in all taxa. A single large lacrimal foramen leading to the lacrimal canal was present on the orbital surface of the lacrimal bone in the clouded leopard and Pallas’s cats. In African and Angola lions, this foramen was located on the orbital margin below the rostral lacrimal process (Fig. 20e and h).

The infraorbital foramen was connected to a short infraorbital canal measuring 4.19 mm (± 0.5 mm) in the clouded leopard, 15.36 mm (± 0.8 mm) in African lions, 17.64 mm (± 0.9 mm) in Angola lions, and 3.14 mm (± 0.2 mm) in Pallas’s cats, and communicated with the maxillary foramen (sphenopalatine surface) (Fig. 20i and l). The infraorbital foramen was located at the level of the second premolar tooth (P2) in the clouded leopard and Pallas’s cat, whereas in African and Angola lions it was located between the third and fourth premolar teeth (P3 and P4) (Fig. 20i and l). On the perpendicular plate of the palatine bone, two openings were present: a smaller caudal palatine foramen in all examined taxa and, directly above it, a larger sphenopalatine foramen in the clouded leopard and Pallas’s cats. In African and Angola lions, the sphenopalatine foramen was much larger and located caudal to the caudal palatine foramen (Fig. 20m and p). A summary of the main comparative observations is provided in Table 10.

**Table 10:** Major differences in the eye morphology and the orbital region

MORPHOLOGIAL CRITERIA	Clouded leopard (Neofelis nebulosa)	Angola lions (Panthera leo bleyenberghi)	Pallas’s cat (Otocolobus manul manul)
AED	medium	largest	smallest
ESE	most	moderate	least
SBV	sparse	sparse	numerous
SM	absent	absent	numerous
CEL	3–4	6–9	7–8
CD	moderate	largest	smallest
BM	absent	absent	absent
ACEL	7–8	11–12	6–8
ACET	thickest	thinnest	moderate
PSCT	moderate	thickest	thinnest
PLMT	moderate	thickest	thinnest
CLBLVM	numerous	sparse	numerous
CLEL	5–7	9–12	3–6
CLP	strong	strong	slight
CAT	moderate	smallest	greatest
CPT	greatest	moderate	smallest
SLT	moderate	greatest	smallest
SET	moderate	greatest	smallest
SONT	greatest	greatest	smallest
PS	round	ovoid	round
CM	absent	numerous	numerous
TLCD	intermediate	largest	smallest
TLT	intermediate	greatest	smallest
ChT	thinnest	thickest	thinnest
ACD	shallower	deeper	shallower
ISM	numerous	numerous	spares
LAL	intermediate	longest	shortest
VCD	moderate	greatest	smallest
EL	intermediate	longest	shortest
AEEL	8–10	6–9	3–5
MZEL	10–15	10–12	4–10
NTG	26–29	35–39	31–33
PEEL	6–8	4–6	5–6
CALT	lower eyelid	lower eyelid	both eyelids
SGTES	intermediate	largest	smallest
SGTESp	round	elongated	round
SGTELb	strong	intermediate and small	intermediate and small
PCEL	6–8	8–10	4–5
BCEL	5–7	5–7	3–4
GCS	sulfated and carboxylated acid mucopolysaccharides and neutral mucins	sulfated and carboxylated acid mucopolysaccharides, sulfated and carboxylated sialomucins (glycoproteins)	sulfated and carboxylated acid mucopolysaccharides and neutral mucins
LGC	thick	thick	thin
LGL	numerous large and small	numerous large and small	several large and few medium and small
LGS	serous	serous	serous
OL	long	long	short
ZPF	short	short	long
SIM	rounded	rounded	sharpened
OTC	not visible	sharp, high, and very well-defined	not visible
EF	double and small	single and large	double and small
PC	weakly marked	well-defined	weakly marked
IFL	P2	P3 – P4	P2
SF	small	large	small

*AED* Axial Eye Diameter, *ESE* Elliptical shape of eye, *SBV* Scleral blood vessels, *SM* Scleral melanocytes, *CEL* Conjunctiva epithelium layers, *CD* Corneal diameter, *BM* Bowman membrane, *ACEL* Anterior corneal epithelium layers, *ACET* Anterior corneal epithelium thickness, *PSCT* Proper substance of the cornea thickness, *PLMT* Posterior limiting membrane thickness, *CLBLVM* Corneal limbus blood and lymphatic vessels and melanocytes, *CLEL* Corneal limbus epithelium layers, *CLP* Corneal limbus pigmentation, *CAT* Corneal axial thickness, *CPT* Corneal peripheral thickness, *SLT* Scleral limbus thickness, *SET* Scleral equator thickness, *SONT* Scleral optic nerve thickness, *PS* Pupil shape, *CM* Chorioid melanocytes, *TLCD* Tapetum lucidum cell diameter, *TLT* Tapetum lucidum thickness, *ChT* Chorioid thickness, *ACD* Anterior chamber depth, *ISM* Iris stroma melanocytes, *LAL* Axial lens length, *VCD* Vitreous chamber depth, *EL* Eyelids length, *AEEL* Anterior eyelids epithelium layers, *MZEL* Marginal zone epithelial layers, *NTG* Number of tarsal glands, *PEEL* Posterior eyelids epithelium layers, *CALT* Conjunctiva-associated lymphoid tissue, *SGTES* Superficial gland of the third eyelid size, *SGTESp* Superficial gland of the third eyelid shape, *SGTELb* Superficial gland of the third eyelid lobation, *PCEL* Palpebral conjunctiva epithelium layers of the third eyelid, *BCEL* Bulbar conjunctiva epithelium layers of the third eyelid, *GCS* Goblet cells secretion, *LGC* Lacrimal gland capule, *LGL* Lacrimal gland lobation, *LGS* Lacrimal gland secretion, *OL* Orbital ligament, *ZPF* Zygomatic process of the frontal bone, *SIM* Supraorbital and infraorbital margin, *OTC* Orbitotemporal crest, *EF* Ethmoid foramen, *PC* Pterygoid crest, *IFL* Infraorbital foramen location, *SF* Sphenopalatine foramen

## Discussion

The results achieved in this study should be interpreted carefully due to the low number of available samples, the lack of clinical ophthalmological examination, and possible post-mortem changes. The first issue was beyond our control, as access to additional individuals of these taxa is very limited. The clinical examinations carried out by the veterinary service of zoological gardens did not include detailed ophthalmological examinations, which reflects routine veterinary management in non-domesticated animals and welfare-related limitations. Therefore, subclinical abnormalities of the eye cannot be excluded and may have influenced some of the observed features. Finally, post-mortem tissue decomposition, which could affect morphological findings, was minimized by the short interval between animal death and sample collection.

Felids inhabiting diverse natural environments rely strongly on vision during hunting and prey capture. Their eyes represent a visual system capable of functioning under a wide range of light conditions [93]. Under bright lighting, visual performance depends mainly on visual acuity determined by retinal structure and ocular optics, as well as on color discrimination. In cats, visual acuity is lower than in humans and some other mammals, likely reflecting retinal limitations rather than the optical system or central processing [94]. The present findings provide a comparative morphological description of selected ocular structures in three felid taxa. However, because of the very limited number of specimens, these observations should be regarded strictly as descriptive and should not be interpreted as species-level patterns. The present material also does not allow conclusions regarding sex-related variation, functional significance, or ecological adaptation. Nevertheless, these data may serve as a cautious point of reference for future studies based on larger and more standardized material. Although post-mortem tissue changes were minimized, their possible influence on morphological and morphometric findings cannot be excluded. Because of the very limited number of specimens, the present study does not allow for thevaluation of species-level anatomical patterns, and all observations should be interpreted strictly as findings in the examined material.

### Eyeball and eye tunics

Eyeball dimensions (AED, MaEED, MiEED) generally corresponded to overall body size. Among the examined individuals, the Angola lions, as the largest taxa represented in the material, had larger eyeballs than the clouded leopard and Pallas’s cat, and a difference between the male and female Angola lion was also observed. Differences between maximum and minimum equatorial diameters indicated variation in globe shape among the examined individuals: the Angola lions had a more elliptical equatorial profile, whereas the clouded leopard presented a nearly round equatorial outline and the Pallas’s cat an intermediate shape. As noted by Walls [93], eyeball size is one factor influencing visual performance, together with retinal ganglion cell distribution. However, the present material does not allow functional interpretation of the observed variation. Unfortunately, the quantitative limitations of the study did not allow any final conclusions. It should also be emphasized that morphometric values obtained from post-mortem and processed material may be affected by tissue deformation, shrinkage, and sectioning, and therefore should be interpreted as approximate descriptive values rather than precise anatomical measurements.

Comparisons with other carnivores indicate that eye size does not scale uniformly with body mass across families and may be influenced by multiple factors, including head size, diet, and activity pattern [94–96]. According to Heard-Booth & Kirk [95], Kirbas Dogan et al. [96], and Paszta et al. [97], studies comparing eyeball size in Ursidae and selected Canidae species showed similar dimensions despite marked differences in body size. In contrast, mixed-breed dogs, South African painted dogs (*Lycaon pictus pictus*), gray wolves (*Canis lupus*), gray foxes (*Urocyon cinereoargenteus*), and red foxes (Vulpes vulpes) showed larger eyeball parameters [95, 97, 98]. The greatest variation in eyeball dimensions within Canidae has been reported in domestic dogs and has been linked to craniometric diversity, skull type, and orbital dimensions [99]. Heard-Booth & Kirk [95] also suggested associations between vertebrate eye size and body or head size, diet, activity pattern, and locomotor speed. These comparative data show that eye size may vary under the influence of multiple factors; however, the present material is too limited to evaluate which of them may be relevant in the examined felids.

When comparing axial eye diameter in the present material with published data for tiger (*Panthera tigris*), African lion, and cheetah [95], the values reported for those species were similar and in some cases larger than in the examined Angola lions. Hollister [100] suggested that captive housing may influence some anatomical dimensions in lions. Castello [101] reported that the Pallas’s cat and clouded leopard have relatively large eyes in relation to body size, and Montiani-Ferreira et al. [102] noted that cats possess large, frontally positioned eyes that support binocular vision. In the context of the present study, these published observations may be useful for comparison, but the available material does not allow direct ecological or behavioral interpretation of the eyeball measurements obtained here.

Corneal measurements also appeared to vary with overall body size, as the examined Angola lions had larger corneal values than the smaller felids. Differences between the male and female Angola lion were also noted. Histologically, the cornea in the examined specimens consisted of four layers, with no anterior limiting membrane (Bowman’s layer), consistent with reports in domestic cats, dogs, wolves, dingoes, and corsac foxes [103–105]. The anterior corneal epithelium showed variation in the number of epithelial cell layers, as also reported among felid species by Merindano et al. [12]. Similar findings have been described in the domestic cat, domestic dog, wolf, dingo (*Canis dingo*), corsac fox (*Vulpes corsac*), and Asiatic black bear (*Ursus thibetanus*) [103, 104, 106]. In the present material, the number of epithelial cell layers was highest in the Angola lions and lowest in the clouded leopard, whereas the Pallas’s cat showed intermediate values. However, given the very limited sample size and the potential influence of individual condition and tissue processing, these observations should be treated as descriptive only. Patruno et al. [107] reported only 3 to 4 epithelial cell layers in the domestic cat, which also indicates that this parameter may vary across studies. At present, the basis of this variation remains unclear in the examined material.

The palisades of Vogt were identified at the corneal limbus, similar to those described in domestic cats, dogs, and humans [108]. These structures are considered a niche for limbal epithelial stem cells involved in corneal surface renewal and maintenance of transparency [108]. In humans, limbal stem cell deficiency may lead to conjunctivalization and vascularization of the cornea [109]. Although the present study has no clinical component, the identification of palisades of Vogt adds comparative anatomical information and may be relevant for future studies of limbal microanatomy in wild felids.

In felids, the tapetum lucidum is regarded as an important component of vision under low-light conditions [9, 83]. In the present material, the Angola lions had the largest tapetum lucidum dimensions, the clouded leopard showed intermediate values, and the Pallas’s cat the smallest. Because only a very small number of individuals was available, these differences should not be interpreted as taxon-level or habitat-related patterns. Tapetum lucidum coloration also differed among the examined individuals (milky-blue vs. blue-green). Tapetal color is known to vary across mammals and may change with age and species, likely reflecting differences in iridocyte structure, refractive indices, and light interaction with the tapetal layer [102]. In domestic cats, the shape and color of the tapetum lucidum are variable, and fundus color may also change with age [102]. The influence of post-mortem changes on tapetal color cannot be excluded. Donisa et al. [110] suggested that tapetum lucidum color may also be associated with coat color and the degree of pigmentation in the eye and skin. In domestic cats, the tapetum lucidum appears semicircular in outline and is typically yellow or orange [111], although Ollivier et al. [14] noted that it may also be triangular and vary in color from yellow to green. Histologically, the examined felids showed a choroidal tapetum lucidum cellulosum, consistent with previous reports in carnivores [9]. Differences in tapetal cell arrangement and thickness were observed among the examined individuals, but the present material does not allow conclusions regarding their functional significance.

Anterior aqueous chamber depth (AACD) also varied among the examined individuals, with greater values in the Angola lions than in the clouded leopard and Pallas’s cat. A relatively larger AACD and a lens positioned farther back in the globe have been associated with low-light vision in felids [112]. However, the present data do not allow assessment of whether the observed differences reflect taxon-related optical patterns or individual variation. Pupil shape varies widely among terrestrial mammals and has been discussed in relation to ecological niche, activity pattern, and optical strategy [113]. In the examined material, the pupil appeared round at rest in the clouded leopard and Pallas’s cat, whereas it was horizontally ovoid in the Angola lions. Because these observations were made post-mortem and the sample size was very limited, they should be interpreted cautiously. In both Angola lions, an iridic granule (corpora nigra) was identified at the dorsal pupillary margin. In domestic species such as horses and cattle, corpora nigra are considered normal anatomical structures that reduce glare and limit excessive light entry [114]. In the absence of veterinary records suggesting ocular disease and without histologic evidence of pathology, these structures are interpreted here as anatomical findings in the examined material rather than as lesions [114].

Lens axial length correlated with overall eyeball size, with the Angola lions showing larger values than the smaller felids. A difference between the male and female Angola lion was also recorded. Larger pupils require proportionally larger lenses to maintain appropriate light transmission and reduce optical aberrations under low light [112, 113, 115, 116]. Nevertheless, the present material is not sufficient to assess these relationships in the examined felids. The above-mentioned dimensional values must be treated carefully due to the small number of examined individuals and possible post-mortem deformation of the eyeball. Moreover, post-mortem changes may influence chamber measurements, corneal thickness, tissue hydration, and measurements taken after sectioning.

Overall, the present findings expand the descriptive anatomical record for the felid eye, but they do not permit conclusions regarding ecological adaptation, visual performance, or species-specific optical strategies. These issues require future investigation using larger series, standardized clinical evaluation, and, where possible, material collected under more uniform conditions.

### Eyelids, conjunctiva, and ocular surface protection

Upper and lower eyelids are essential protective structures and contribute to tear film maintenance and drainage [117–119]. Eyelashes were present only on the anterior palpebral margin of the upper eyelid, consistent with reports in domestic cats and several carnivores [83, 97, 98]. Morphometric differences among the examined individuals may reflect differences in overall body size; however, the present material does not allow for further interpretation of their functional or environmental significance. Accordingly, these observations should be treated as descriptive only. Further studies based on larger series are needed before any broader conclusions can be drawn.

Histologically, the eyelids in the examined material were generally comparable to those reported in domestic cats, dogs, and other carnivores, with differences in epithelial organization and glandular distribution that were visible among the examined individuals and were consistent in part with previous descriptions [83, 98]. The conjunctival epithelium included goblet cells, supporting mucin production in the precorneal tear film and contributing to ocular surface protection [120, 121]. Importantly, in cats with clinically and histologically normal conjunctiva, goblet cell density and distribution vary significantly between conjunctival regions; therefore, the sampling site has a direct impact on the interpretation of goblet cell findings [122, 123]. As reported by Davidson & Kuonen [120], Knop & Knop [121, 124], Moore et al. [125], and Moore & Tiffany [18], the density of goblet cells in the bulbar conjunctiva of the eyelids differs between species and also depends on the site sampled and the condition of the tissue examined. In the present study, the absence of detailed ante-mortem ophthalmological examination further limits interpretation of these findings.

Conjunctiva-associated lymphoid tissue elements were also identified, consistent with findings in domestic cats, dogs, and other carnivores, and indicating the presence of local immune tissue at the ocular surface [97, 98]. Such structures are considered physiological and may respond to environmental antigen exposure and infectious challenges [83]. However, because of the limited material, the present study does not allow assessment of the degree to which the observed CALT components reflect taxon-related differences, individual variation, or local tissue condition.

### Third eyelid, superficial gland of the third eyelid, and lacrimal gland

The superficial gland of the third eyelid (SGTE) was located at the medial canthus in all examined felids, consistent with domestic cats and other carnivores [97, 126]. SGTE size appeared to correspond broadly to overall body size, with the largest glands in the Angola lions and the smallest in the Pallas’s cat. However, because of the very limited material, no final conclusion can be drawn regarding this relationship. Although Lantyer-Araujo et al. [98] and El-Naseery et al. [127] reported differences in lacrimal gland dimensions in domestic dogs related to breed, sex, and skull type, the limited number of specimens available in the present study does not allow comparable interpretation in the examined felids. Histologically, SGTE showed a multilobular compound branched acinar organization with serous secretion, similar to that described in the domestic cat and South African painted dog [97, 128]. In contrast, a seromucous SGTE has been described in domestic dogs [83]. Serous secretion provides water, electrolytes, proteins, and enzymes important for ocular surface defense and maintenance [129]. In the present material, both SGTE and the lacrimal gland showed predominantly serous features. However, the available specimens do not allow assessment of whether this reflects a broader felid pattern.

The third eyelid (TE) showed a typical T-shape, consistent with domestic cats and other carnivores [83, 97, 98, 130, 131]. In the examined felids, TE cartilage was elastic, similar to that reported in domestic cats and the Indian mongoose, and distinct from the hyaline cartilage reported in Canidae [83, 97, 128, 131, 132]. The present study documents this structural pattern in the examined material, but its mechanical or functional significance was not assessed. Lymphoid follicles were present within the TE conjunctiva, consistent with prior descriptions and supporting the descriptive presence of CALT-related tissue at the ocular surface [97, 98, 130].

The lacrimal gland (LG) was located in the dorsolateral angle of the globe, as in domestic cats and other carnivores [83, 97, 131]. Among the examined individuals, the Angola lions had larger LG dimensions than the smaller felids; however, the number of specimens was too limited for further interpretation. Histologically and histochemically, LG showed a multilobular acinar structure producing serous secretion, consistent with reports in Canidae [83, 97, 131], although structural variation has been described in domestic dogs [127]. Together with SGTE, goblet cells, and eyelid glands, LG contributes to the tear film covering the ocular surface and is associated with hydration, nutrition, waste removal, particle clearance, and antimicrobial defense [133, 134]. The present findings extend the descriptive anatomical record of the lacrimal apparatus in wild felids, but they do not allow conclusions regarding taxon-level patterns or environmental associations.

### Orbital region

The examined felids showed an open orbit formed by bony structures comparable to those described in domestic cats, dogs, and other carnivores [16, 97]. Selected osteological differences were observed among the examined skulls, including variation in the ethmoid foramina and in the prominence of certain crests and processes, consistent with published descriptions of felid cranial diversity [52]. These observations indicate that orbital osteology may vary within the examined material; however, the present sample size is too limited to determine whether these differences represent broader taxon-level patterns. Further study of a larger series would be required to evaluate this issue more reliably. 

The observed differences should therefore be interpreted as individual findings within the examined material and not as representative traits of the studied species.

## Conclusions

Due to the limited number of individuals accessible for this study, these conclusions are necessarily preliminary and should be interpreted cautiously. Nevertheless, the comparative gross and histological observations indicate that several ocular and adnexal features were present in the examined material and were broadly comparable with previous descriptions in the domestic cat. They include the key elements of corneal organization, the presence of limbal structures supporting epithelial renewal, and a generally similar architecture of the lacrimal apparatus and conjunctival protective components. At the same time, differences in the absolute size and selected proportions of the eyeball and ocular adnexa were observed among the examined individuals; however, because of the very limited sample size, these findings should be interpreted strictly as descriptive observations and not as species-level patterns. Further studies are needed before broader conclusions can be drawn. Collectively, these findings provide a comparative anatomical baseline for understudied wild felids and may support future comparative studies based on larger and better-characterized samples. The clinical relevance of this study may also be noted. Practical knowledge in zoological ophthalmology, orbital surgery, and diagnostic imaging interpretation remains limited. Anatomical studies of the eyeball in wild carnivores may contribute to clinical veterinary medicine by expanding comparative morphological knowledge and by providing a better description of the architecture of the eye and adjacent structures.

### Limitations

This study is primarily limited by specimen quantity. Ocular and ocular adnexal analyses were based on four individuals representing three felid taxa (including two *Panthera leo bleyenberghi*), which restricts the work to descriptive, comparative observations and precludes population-level inference, robust assessment of inter-individual variability, and formal testing of sex- or age-related effects within taxa. Although repeated measurements were performed to improve technical precision, these repeats do not represent independent biological replicates. Additional limitations relate to specimen heterogeneity and incomplete metadata. The examined animals differed in age and sex and originated from zoological collections; therefore, potential influences of captivity, husbandry, and individual life history cannot be excluded. Ante-mortem ophthalmic examinations were not done. While veterinary records and post-mortem inspection did not indicate gross ocular pathology, subclinical disease cannot be ruled out. Both clinical and subclinical pathologies may influence the morphological status of analyzed structures, which limits interpretation of ocular normality. Methodological constraints inherent to post-mortem material should also be considered. Despite a short post-mortem interval, tissue handling and fixation may introduce artifacts (e.g., shrinkage or hydration-related changes) that can affect morphometric values and histological appearance. In addition, because some ocular structures were measured after dissection, fixation, or sectioning, partial deformation or collapse of delicate tissues cannot be excluded. Therefore, the morphometric values presented here should be regarded as descriptive measurements of the examined material rather than as absolute species-level reference standards. Post-mortem changes may also influence macroscopic features such as tapetal coloration and should be considered when interpreting color-related observations. Finally, orbital morphometry additionally relied on skull collections obtained from different sources; background information was incomplete for some specimens, and cranial damage precluded selected measurements. For a subset of lions, missing body and head measurements necessitated the use of literature-derived values, which may reduce comparability across specimens.

### Future recommendations

Future studies should prioritize larger, standardized cohorts within each species (and, where relevant, subspecies), with balanced age and sex representation to enable assessment of inter-individual variability and more reliable evaluation of whether any broader taxon-related patterns are present. Whenever feasible, standardized post-mortem intervals, collection procedures, and fixation protocols should be implemented, and detailed metadata (origin, husbandry history, clinical history, and cause of death) should be recorded to strengthen interpretation and reproducibility. Integrating ante-mortem ophthalmic assessment would substantially improve interpretation of normal versus potentially altered ocular morphology. Clinical examination and imaging (e.g., ultrasound, CT/MRI where appropriate), and when available, living-animal modalities such as optical coherence tomography, would facilitate linkage between gross/microscopic anatomy and ocular function and health status. For orbital analyses, expanding well-documented skull series and applying three-dimensional approaches (CT-based 3D reconstructions and geometric morphometrics) would provide a more robust comparative framework for felids. Finally, transparent reporting of raw measurements and imaging documentation (including clear measurement definitions and calibration procedures) would improve cross-study comparability and support future meta-analytic work. Potential functional or ecological interpretation of ocular morphology should be addressed only after analysis of substantially larger and more standardized material.

## Supplementary Information

Supplementary Material 1

## Data Availability

Material available by reasonable request to the corresponding author (j. [kleckowska-nawrot@upwr.edu.pl] (mailto: kleckowska-nawrot@upwr.edu.pl) ).
