# Supplementary material for: Descriptive comparative morphology of the eye and ocular adnexa in Felidae: observations from a clouded leopard (Neofelis nebulosa), African lion (Panthera leo), and Pallas’s cat (Otocolobus manul)
Source: BMC Vet Res. 2026 Apr 22;22:329. doi: 10.1186/s12917-026-05480-1 (PMC13238145; doi:10.1186/s12917-026-05480-1)
Supplement: Supplementary file 1 — Supplementary Material 1 [file 12917_2026_5480_MOESM1_ESM.docx]

**Table S1**

Full literature overview table (Supplementary).

| **SPECIES** | **MORPHOLOGY** | | | | | **OPHTHALMOLOGICAL EXAMINATION AND PATHOLOGY** | **LITERATURE** |
| --- | --- | --- | --- | --- | --- | --- | --- |
|  | **ORBITAL REGION/ EYE TUNICS** | **UPPER and LOWER EYELIDS** | **SUPERFICIAL GLAND OF THE THIRD EYELID** | **THIRD EYELID** | **LACRIMAL GLAND** |  |  |
| **FELIDS – FELIDAE** | | | | | | | |
| **African golden cat** (*Caracal aurata* Temminck, 1827) | + (cornea) | – | – | – | – | – | [12] |
| **Black-footed cat** (*Felis nigripes* Burchell, 1824) | – | – | – | – | – | + | [31] |
| B**obcat** (*Lynx rufus* Schreber, 1777) | + (retina) | – | – | – | – | + | [19, 32] |
| **Caracal** (*Caracal caracal* Schreber, 1776) | – | – | – | – | – | + | [19, 33] |
| **Cheetah** (*Acinonyx jubatus* Schreber, 1775) | + (cornea) | – | – | – | – | + | [12, 32, 34–41] |
| **Cougar** (*Puma concolo*r Linnaeus, 1771) | + (cornea) | – | – | – | – | + | [12, 42–44] |
| **Domestic cat** (*Felis catus* Linnaeus, 1758) | + (choroid, cornea, tapetum lucidum, retina, nerves of eye and ocular adnexes, electroretinography) | + | + | + | + | + | [45–50] |
| **Savannah cat** (*Felis catus*) | – | – | – | – | – | + | [19] |
| **Eurasian lynx** (*Lynx lynx* Linnaeus, 1758) | – | – | – | – | – | + | [19] |
| **Fishing cat** (*Prionailurus viverrinus* Bennett, 1833) | + (electroretinograhy) | – | – | – | – | + | [19, 51] |
| **Flat-headed cat** (*Prionailurus planiceps* Vigors & Horsfield, 1827) | +  (orbital region – orbit type) | – | – | – | – | – | [52] |
| **Geoffroy’s cat** (*Leopardus geoffroyi* d'Orbigny & Gervais, 1844) | – | – | – | – | – | + | [19] |
| **Iberian lynx** (*Lynx padinus* Temminck, 1827) | – | – | – | – | – | + | [19] |
| **Jaguar** (*Panther onca* Linnaeus, 1758) | – | – | – | – | – | + | [19, 53, 54] |
| **Jungle cat** (*Felis chaus* Schreber, 1777) | – | – | – | – | – | + | [27, 55] |
| L**eopard** (*Panthera pardus* Linnaeus, 1758) | + (cornea, retina) | – | – | – | – | + | [12, 19, 32] |
| **Leopard cat** (*Prionailurus bengalensis* Kerr, 1792) | + (electroretinograhy) | – | – | – | – | + | [19, 51] |
| **Marblet cat** (*Pardofelis marmorata* Martin, 1837) | +  (orbital region – orbit type) | – | – | – | – | – | [52] |
| O**celot** (*Leopardus pardalis* Linnaeus, 1758) | – | – | – | – | – | + | [19] |
| **Sand cat** (*Felis margarita* Loche, 1858) | – | – | – | – | – | + | [19] |
| **Serval** (*Leptailurus serval* Schreber, 1776) | – | – | – | – | – | + | [19] |
| **Snow leopard** (*Panthera uncia* Schreber, 1775) | – | – | – | – | – | + | [19, 56–66] |
| **Tiger** (*Panthera tigris* Linnaeus, 1758) | + (cornea) | – | – | – | – | + | [12, 18, 19, 67–69] |
| **Bengal tiger** (*Panthera tigris* spp t*igris* Linnaeus, 1758) | – | – | – | – | – | + | [70–72] |
| **Siberian tiger** (*Panthera tigris altaica* Temminck, 1844) | – | – | – | – | – | + | [73, 74] |
| **HYAENAS – HYAENIDAE** | | | | | | | |
| **Spotted hyena** (*Crocuta crocuta* Erxleben, 1777) | + (retina) | – | – | – | – | – | [75] |
| **Striped hyena** (*Hyaena hyaena Linnaeus*, 1758) | – | – | – | – | – | + | [76] |
| **MONGOOSE – HERPESTIDAE** | | | | | | | |
| **Angolan kusimanse** (*Crossarchus ansorgei* Thomas, 1910) | + (retina) | – | – | – | – | – | [77] |
| **Small Indian mongoose** (*Herpestes auropunctatus* Hodgson, 1836) | + | – | – | + | – | – | [78, 79] |
